# A Study of the Infant Nasal Microbiome Development over the First Year of Life and in Relation to Their Primary Adult Caregivers Using *cpn60* Universal Target (UT) as a Phylogenetic Marker

**DOI:** 10.1371/journal.pone.0152493

**Published:** 2016-03-28

**Authors:** Shelley W. Peterson, Natalie C. Knox, George R. Golding, Shaun D. Tyler, Andrea D. Tyler, Philip Mabon, Joanne E. Embree, Fiona Fleming, Sergio Fanella, Gary Van Domselaar, Michael R. Mulvey, Morag R. Graham

**Affiliations:** 1 Department of Medical Microbiology, University of Manitoba, Winnipeg, Manitoba, Canada; 2 Bacteriology Division, National Microbiology Laboratory, Public Health Agency of Canada, Winnipeg, Manitoba, Canada; 3 Science Technology Cores & Services Division, National Microbiology Laboratory, Public Health Agency of Canada, Winnipeg, Manitoba, Canada; 4 Department of Pediatrics and Child Health, University of Manitoba, Winnipeg, Manitoba, Canada; 5 Pediatric Urgent Care, Manitoba Clinic, Winnipeg, Manitoba, Canada; 6 Department of Computer Science, University of Manitoba, Winnipeg, Manitoba, Canada; National Centre for Cell Science, INDIA

## Abstract

Whereas the infant gut microbiome is the subject of intense study, relatively little is known regarding the nares microbiome in newborns and during early life. This study aimed to survey the typical composition and diversity of human anterior nare microflora for developing infants over time, and to explore how these correlate to their primary caregivers. Single nare swabs were collected at five time points over a one-year period for each subject from infant-caregiver pairs. Our study comprised of 50 infants (recruited at 2 weeks, post delivery) and their 50 primary caregivers. Applying the chaperonin-60 (*cpn60)* universal target (UT) amplicon as our molecular barcoding marker to census survey the microbial communities, we longitudinally surveyed infant nares microbiota at 5 time points over the course of the first year of life. The inter- and intra-subject diversity was catalogued and compared, both longitudinally and relative to their adult primary caregivers. Although within-subject variability over time and inter-subject variability were both observed, the assessment detected only one or two predominant genera for individual infant samples, belonging mainly to phyla Actinobacteria, Firmicutes, and Proteobacteria. Consistent with previously observed microbial population dynamics in other body sites, the diversity of nares microflora increased over the first year of life and infants showed differential operational taxonomic units (OTUs) relative to their matched primary caregiver. The collected evidence also support that both temporal and seasonal changes occur with respect to carriage of potentially pathogenic bacteria (PPBs), which may influence host predisposition to infection. This pilot study surveying paired infant/caregiver nare microbiomes provides novel longitudinal diversity information that is pertinent to better understanding nare microbiome development in infants.

## Introduction

Under normal circumstances, an estimated 10^14^ commensal bacterial cells live within the human body, easily outnumbering human cells [1,2]. Scientists increasingly recognize that humans are superorganisms colonized by thousands of microbial species harbouring at least 100 times as many genes as our own [3], and this microbial population plays an important role in health and disease. With the advent of cost effective, high throughput molecular methods, a number of studies aiming to determine the compositions of microbial communities that colonize humans have been conducted. Studies to date have revealed human-associated microbiota are diverse across different anatomical sites, vary between individuals, and can vary temporally within individuals [4,5]. Healthy microbiota are primarily considered benign or even beneficial to the host, by physically protecting against colonization by invading pathogens, stimulating host innate immune defenses, aiding tissue and immune system development, and promoting drug metabolism [6–11]. However, when microbial communities within niche anatomical sites are perturbed, these normally symbiotic relationships can undergo disruptive imbalance (dysbiosis) that may ultimately lead to immune dysregulation and/or susceptibility to disease [2,12]. Consequently, microbiome surveys are central to better understanding human health and disease.

The Human Microbiome Project (HMP) and other early microbiota surveys have revealed the respiratory tract harbours very diverse microbes [12–14]. The anterior nare (nostril) represents a highly accessible airway microbial community, which can serve as a reservoir for opportunistic microbial pathogens that may then seed infections elsewhere. For example, *Corynebacterium*, *Propionibacterium*, *Staphylococcus*, and *Moraxella* are bacterial pathogens routinely and predominantly detected in healthy adult nares [13,15,16]. In conjunction with increased risk for transmission of persistent/colonizing antimicrobial resistant (AMR) strains, asymptomatic nasal carriage is associated with increased risk for acute respiratory illnesses of both the upper and lower respiratory tracts, skin and soft tissue infections, and bacteremia [17,18]. Consequently, there is pressing need to better detail the microbial ecology of the nares, including ecological factors influencing the composition, diversity, dynamics, and function of this human-associated microbial community.

Before birth, the infant resides within the sterile environment of the uterus. The infant first encounters microorganisms upon entry into the vaginal canal during natural birth, or via contact with skin in the case of caesarean section [19]. Colonization with microflora occurs within the first few hours of life through respiration, ingestion, and contact of skin to foreign surfaces [2,20]. In addition, microorganisms are transferred from the mother to the infant during breastfeeding [21,22]. Studies of the gut microbiome have revealed that neonatal microbiota have low diversity at birth; afterward increasing over the first several months of life. Infant microbiomes are dynamic, with high intra-individual variability until around 1–3 years of age, when a more stable, adult-like microbiome becomes established [22–25].

Few studies have surveyed microflora of infant human nares. Bogaert *et al*. tested nare swabs from infants aged 18-months, determining that roughly 80% of detected organisms were Gram-positive (predominantly Actinobacteria and Firmicutes) [26]. Owing to high interpersonal variability for the identified taxonomic groups, the study concluded that a few common “core” organisms could be said to make up a nares “core” microbiome [8]. Current understanding is also limited for how microflora varies over time (*e*.*g*., microbiome dynamics) in healthy infants. Thus, we designed this paired cohort study (comprised of infants and matched caregivers) as a pilot study to explore such knowledge gaps. Applying the chaperonin-60 (*cpn60)* universal target (UT) amplicon as our molecular barcoding marker to census survey the microbial communities, we longitudinally surveyed infant nares microbiota at 5 time points over the course of the first year of life. The inter- and intra-subject diversity was catalogued, and compared both longitudinally and relative to their adult primary caregivers. This study enhances our knowledge of the ranges of normal variability in human nares microbiome diversity and community structure and provides insights into potential ecological factors influencing developmental dynamics in the first year of life. Baseline knowledge of nares microbiome development and its influencing factors are important, as it may assist us in better recognizing abnormal situations and prevent disease in a highly vulnerable population.

## Materials and Methods

### Ethics statement

This study was approved by the institutional Research Ethics Boards of both the University of Manitoba (Reference #H2009:206) and of Health Canada (Reference #2009–0021 with appropriate informed consent and coded specimens following the guidelines of the approved protocol.

### Study design and subjects

Fifty infants and their primary caregivers were recruited from the Manitoba Clinic (Winnipeg, Manitoba, Canada) during their first post-natal appointment, when infants were two weeks of age. A written consent form was reviewed during the first clinic visit by each infant’s adult caregiver. Written consent to participate in the study was provided on behalf of both study participants via signature by each adult. The nares of infants and primary caregivers were sampled by a study paediatrician 5 times throughout a one year period (T1-T5), with sampling time points corresponding to infant ages: 2-weeks (T1, January-February), 2-months (T2, March-April), 4-months (T3, May-June), 6-months (T4, July-August), and 1-year (T5, end of January to beginning of April). At each clinic visit, each paired participant set (infant/caregiver) filled in a written study questionnaire to acquire associated information about living conditions, general health, smoke exposure, infant delivery method, antibiotic use, and infant feeding method (S1 File).

### Nares sampling and DNA preparation

A non-invasive sample collection protocol was developed based on recommendations contained within the Human Microbiome Project (HMP) Core Microbiome Sampling Protocol A [27]. At each clinic visit (sampling time points T1 through T5), one sterile flocked nylon swab (FLOQSwab™; Copan Diagnostics Inc., Murrieta, CA, USA) was used per subject to swab one of the anterior nares. Each swab was then placed into a 1.5 ml Eppendorf^®^ DNA LoBind tube (Eppendorf Canada, Mississauga, Canada) containing 500 μl of Oragene^®^-DNA chaotropic preservant solution (DNA Genotek, Kanata, Canada).

The MolYsis™ Basic kit enrichment (Molzym Life Science, Bremen, Germany) was applied to these low-biomass swabs to selectively lyse human cells and degrade non-target human DNA prior to DNA template extraction. Both 50 μl buffer DB1 and 10 μl MolDNase A were added to each tube containing the Oragene^®^-nasal swab suspension and incubated at room temperature for 15 min. The “V”-bottoms of 0.6 ml microcentrifuge tubes were cut off with sterile scissors, the tubes placed into 1.5 ml collection microtubes, and the processed swabs were placed inside the 0.6 ml open-bottom microtubes. These microtube constructions were centrifuged twice for 5 min at 800 x g to collect all swab liquid in the collection tubes. Swabs were then discarded and the remainder of the MolYsis™ target enrichment protocol was performed on the eluants as per manufacturer instructions.

The microbe-enriched DNA templates were purified applying the Agencourt Genfind™ v2 Blood & Serum DNA Isolation Kit as per the manufacturer’s protocol (Beckman Coulter Genomics, Danvers, USA). Thereafter, the low-biomass templates were subjected to linear augmentation by multiple displacement amplification (MDA) in duplicate using the GenomiPhi v2 kit (GE Healthcare Life Sciences, Baie d’Urfe, Canada). Residual reaction components were removed using the MinElute™ PCR purification kit (Qiagen, Toronto, Canada). To concentrate the eluants, each duplicate reaction was applied to a single MinElute column and co-eluted with 25 μl of warmed EB buffer (10mM Tris-Cl pH 8.5).

### Amplification of the *cpn60* universal target

A set of custom-designed LibL-*cpn60* UT amplicon fusion primers (Table A in S2 File) were manufactured in-house based on existing P279/P280 and P1612/P1613 *cpn60* UT primer pairs [28,29], and in accordance with amplicon fusion primer design guidelines for GS FLX Titanium Series Lib-L chemistry (Roche Applied Science). These primer sets included internal Multiplex Identifier (MID) tag sequences for each subject (Table B in S2 File) to enable automated software identification of samples after pooling/multiplexing and pyrosequencing. Amplification of the LibL-adapted *cpn60* UTs was conducted in nine PCR reactions per sample, as per the PCR reaction conditions described in the supplementary methods (Text Preamble in S2 File) [30]. Triplicate PCR reactions were pooled together and purified using the AMPure XP purification system (Agencourt Bioscience, Danvers, USA), as per manufacturer instructions applying 0.7x AMPure XP, and eluting templates in a final 80 μl elution volume. Each of three AMPure XP-purified pools (each comprised of triplicate PCR reactions) were combined in equi-volume amounts to generate a combined pool of the nine original PCR amplicons acquired per study subject for each single time point.

### 454 pyrosequencing and data processing

Segregating all of the AMPure XP-purified MID-tagged amplicons from each subject into working groups of 22 unique MID tags, 6 μg of each individual subject’s amplicon pool was pooled into their appropriate MID-working group. Each resultant nares *cpn60* UT amplicon working group (comprised of 22 tags per run) was then pyrosequenced using the GS FLX+ XLR70 sequencer (Roche Applied Science, Laval, Canada). Pyrosequencing was performed unidirectionally from the 5' end of each Adaptor A-tagged amplicon using 200 flows of each nucleotide (ordered TCAG), as per the instrument standard operating procedure. Raw data was processed using gsRunProcessor v2.6 using the default amplicon settings.

### Bioinformatics quality assurance and taxonomic assignment

Published best practices were used as guidelines [31]. Sequence reads were processed and analyzed using *mothur* v1.27.0 as per the *mothur* 454 SOP (Accessed December, 2012) [32], with modifications. Briefly, steps for sequence data curation and downstream analysis using *mothur* were performed as follows: The *PyroNoise* algorithm was used to differentiate between noise generated as a result of the pyrosequencing process and actual diversity found within a sample [33]. Primer sequences and barcodes were removed and reads meeting any of the following parameters were discarded: less than 150 bp in length, containing any ambiguous bases, greater than 2-nt difference away from the custom primer sequence or the MID-barcode sequence, or harbouring homopolymers greater than 8 nt in length.

For taxonomic profiling, the remaining quality-filtered (QF) sequence reads were aligned to a manually curated reference *cpn60* UT sequence alignment generated using ClustalW with all available bacterial *cpn60* UT reference sequences (Version 20121004, accessed December 2012) from the curated cpnDB sequence database (http://www.cpndb.ca) [28,34] using the *align*.*seqs* command in *mothur* [35]. Aligned QF reads were further filtered to exclude sequences not overlapping the same alignment space and trimmed to remove any alignment overhangs. To minimize potential sequencing errors, aligned QF reads were pre-clustered to merge them with other highly similar QF reads (within 2 bp similarity) using the pre.cluster command in *mothur* [35]. PCR chimera detection and removal was performed using *mothur*’s implementation of UCHIME [36]. A distance matrix was generated using the QF aligned reads and clustered into operational taxonomic units (OTUs) with the average neighbour algorithm at a genetic distance cutoff of 97% identity. Samples with fewer than 1000 reads were excluded from downstream analysis. OTUs were taxonomically classified using the taxonomy included with the *cpn60* database.

### Statistics

Alpha (α)-diversity and relative taxonomical abundances are reported as the mean with standard deviation. Relative abundances were normalized as the count divided by the total number of sequences per sample. The following α-diversity indices were calculated for each sample: Shannon, Chao1, and Good’s estimation of coverage. Rarefaction curves also were generated. Beta (β)-diversity was measured and assessed between comparator groups (e.g., by time point; also for infant versus caregiver) using the Morisita-Horn diversity index. A phylogenetic tree was generated and viewed using the *tree*.*shared* command in *mothur* (parameters: calc = morisitahorn, subsample = 25000). ‘Carriage’ of PPBs was qualitatively assessed based on the frequency of OTU presence amongst study subjects. Significance testing for differential taxonomic prevalence and carriage frequency was calculated applying Wilcoxon Signed-Rank Tests and McNemar’s Exact Tests in the R v3.0.1 statistical computing environment, as well as *mothur’s* implemenatation of *metastats* [37,38]. OTUs that differed in abundance had *p* and *q* values below 0.05 and were required to have a mean abundance greater than 0.1% in at least one of the communities. Significance values were adjusted for multiple comparisons applying the false discovery rate (FDR) method [39]. Multivariate analysis was performed in R v3.0.1 using linear mixed effects analysis and post hoc Tukey’s tests following arcsine square root transformation of the data using Microsoft Excel.

### Nucleotide sequence accession numbers

The *cpn60* UT sequencing data and metadata generated in this study have been submitted to the NCBI Sequence Read Archive (SRA; http://www.ncbi.nlm.nih.gov/sra/) under Bioproject SRP PRJNA284746 and accession numbers SAMN03768558-SAMN03768853 as per the STROBE checklist (S3 File).

## Results

### Study participants

Of the fifty infants and primary caregivers originally enrolled in the study, eight participant pairs did not complete the study. An additional two subject pairs were excluded owing to limited numbers of reads generated (<1000) for all samples received. The final paired (infant-caregiver) cohort (*n* = 40) was comprised of 25 male and 15 female infants; all primary caregivers were female (S1 Fig). Thirty (75%) infants were born via vaginal delivery; 10 (25%) were delivered by caesarean section. All participants received the National Advisory Committee on Immunization (NACI) and Manitoba Health recommended vaccinations at each clinic appointment [40,41]. At 2 weeks (T1), 48% of infants were exclusively breastfed, while 42% were breastfed with formula supplementation, and 9% were exclusively formula fed. By 4-months of age (T3), 38% of infants were exclusively breastfed, 41% were exclusively formula fed, and 21% were fed a combination of breast milk, formula, and solid foods. At age 6-months (T4), 56% of infants were fed solid foods in addition to breast milk (30%), or formula (20%), or both (6%); while 30% of infants were exclusively formula fed and 12% were exclusively breastfed. By 1 year of age (T5), 91% of infants were fed solid foods: exclusively (36%), or in addition to breast milk (23%), formula (27%), or both (5%). No caregivers had reported chronic respiratory tract diseases in their infants. Infant antibiotic use also was caregiver-reported as follows: one infant received Cephalexin prior to the 2-month time point; two infants received antibiotics prior to the 4-month time point (amoxicillin; the other unknown); two infants prior to the 6-month time point (amoxicillin, the other unknown), and three infants prior to the 12-month time point (penicillin; azithromycin, the latter unknown). No infants were enrolled in daycare before they reached 12-months of age; thereafter, 14 infants (41%) attended daycare. Eight of 40 (20%) primary caregivers were smokers, but all infants were reported to be “unexposed to secondary smoke”.

### Read quality assessment and *cpn60* sequence analysis

Across all cohort samples, 2,969,857 pyrosequencing reads were obtained from three combined instrument runs. After read quality filtering (QF; for quality scores, read length, base call ambiguity, homopolymers) and removal of chimeric reads in *mothur*, the final study data set contained a total of 1,512,776 QF reads. Of these, 1,470,543 (97.2%) high quality sequences were identified via sequence identity as bacterial *cpn60* UT. The majority of excluded reads were removed owing to insufficient read length, given that *cpn60* UT read lengths less than 150 nt cannot be used to distinguish between bacterial species [30].

Rarefaction curves were generated using *mothur* in order to estimate species richness for each study participant at each time point (*i*.*e*., for each independent sample). One infant’s sample was excluded from further study, as it was observed to be an extreme outlier containing nearly double the estimated operational taxonomic units (OTUs) relative to any other single study sample. The average Good’s coverage value was 99% (range: 94.4%-99.8%), implying that a sampling depth of approximately 1000 reads per sample should be sufficient to assess the true biodiversity of each specimen with confidence. Consequently, owing to insufficient depth of coverage deemed requisite for extrapolating phylogenetic diversity, samples with fewer than 1000 QF reads were removed prior to downstream analyses. This left 297 remaining individual samples, originating from the 40 study subject pairs (infants and caregivers).

### Nares microbiome composition

**Fig 1:**
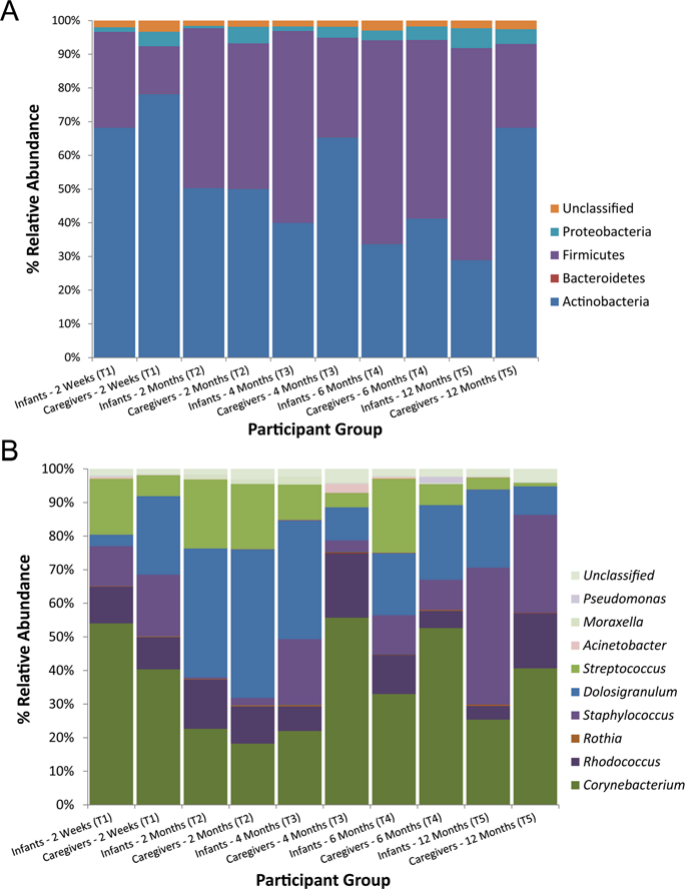
**Average relative abundance of phyla (A) and genera (B) in the nares microbiota of infants and their associated primary caregivers across five time points during the first year of life.** Relative abundance depicted represents the average values derived from each subject grouping at each sampling time point T1-T5 (range: 22–35 subjects per time point, in a possible maximum *n* = 40 study subject pairs).

Across all nares samples, the 1,470,543 high quality *cpn60* UT sequences were classified into 7,758 OTUs, with 2.2% remaining unclassified below the phylum level. Taxonomic classification of OTUs indicated dominant phyla for both infant and adult groups were Actinobacteria (mean 46.8%, SD 37.7) and Firmicutes (mean 45.5%, SD 38.3), with smaller amounts of Proteobacteria (mean 5.4%, SD 14.9), Bacteroidetes/Chlorobi group (observed in low abundance: <1.5% abundance in 7–13% of subjects per time point), and Deinococcus-Thermus (observed in very low abundance: 3 subjects during time points T2, T3 and T5) (Fig 1A). Inter-subject variability was noted, with individual subjects containing a high proportion of either Actinobacteria or Firmicutes, and only a few subjects with a high proportion of Proteobacteria (S2 Fig).

Within the infant-caregiver cohort, we detected dominant taxa previously reported to commonly inhabit the nasal cavity, including Actinobacteria (genera *Corynebacterium*, *Rhodococcus*, and *Propionibacterium*); Firmicutes (mostly genera *Staphylococcus*, *Streptococcus*, *Dolosigranulum* and *Veillonella*), and Proteobacteria (mostly genus *Moraxella*) (Fig 1B). The nares microbiomes were dominated by 6 genera, in rank order: *Corynebacterium* (present in at least 94% of study subjects per time point), *Streptococcus* (≥84%), *Staphylococcus* (≥81%), *Dolosigranulum* (≥66%), *Rhodococcus* (≥59%), and *Rothia* (≥26%), (Tables A and B in S4 File). At any one time point, sampled individuals appeared to carry only one or two predominant genera in their nares; the majority of other taxa were found in much lower relative abundance (S3 Fig). Three relatively common OTUs could not be resolved to identify taxa lower than the taxonomic rank of class or order: namely Actinobacteria (class) (≥59% of study subjects per time point), Gammaproteobacteria (class) (≥25%); and Actinomycetales (order) (≥41%) likely due to insufficient length of sequences for full discrimination, or limited depth of the cpnDB database.

### Comparison of infant and caregiver anterior nare microbiomes

The majority of infants carried as predominant genera one or more of *Corynebacterium*, *Streptococcus*, *Staphylococcus*, and *Dolosigranulum* after T1. Albeit in low abundance, generally adults were more likely to carry *Propionibacterium* (4.5% (infants) *vs*. 38.7% (adults), *p* = 8.8 x 10^−5^, McNemar Exact Test) (S4 File); significantly different OTU relative abundance between infants and caregivers was noted at three time points: T3 (4-months, *p* = 0.00024), T4 (6-months, *p* = 0.00024), and T5 (12-months, *p* = 0.002). *Staphylococcus* also was found more frequently in caregivers than infants (significant at T4 (*p =* 0.0034); and with different OTU relative abundance at the 4-month sampling point (T3–0.4% infants, 7% caregivers; *p* < 0.00001, *q* < 0.0001). Actinobacteria (could not be classified below class level) also was found more frequently in caregivers *vs*. infants at T3 (*p* = 0.0018); and with different OTU relative abundance at the 12-month sampling point (T5–0.2%, infants, 31% caregivers; *p* < 0.00001, *q* < 0.00001). Lastly, with additional rudimentary multivariate investigation also considering time, *Propionibacterium* (*p* = 1.21 x 10^−4^), *Dolosigranulum* (*p =* 9.30 x 10^−4^), and *Streptococcus* (*p* = 3.61 x 10^−6^), as well as class Actinobacteria (*p* = 3.63 x 10^−4^), showed differential relative abundance in infants *vs*. primary caregivers (data not shown). Overall, the data suggest that the predominant phyla and genera of infant nares did not correlate to those of their primary caregivers (S3 Fig). At a genetic distance of 0.03 (roughly somewhere between genus and species level based on *cpn60* UT), 11.7–14% OTUs were shared between infants and caregivers at any given time point (S4 Fig).

Rudimentary multivariate analysis also was attempted to visualize which metadata variables may influence microbiome composition. Likely owing to limited power due to the small sample size in this analysis, few significant differences were found; however *Dolosigranulum* carriage appeared to be affected by size of household (≤4 *vs*. ≥5, *p* = 5.8 x 10^−4^), and in infants the observed abundance of *Staphylococcus* trended higher for infants that were delivered vaginally *vs*. by caesarean section (*p* = 0.06).

### Trending of potentially pathogenic bacteria in the nares microbiome

**Fig 2:**
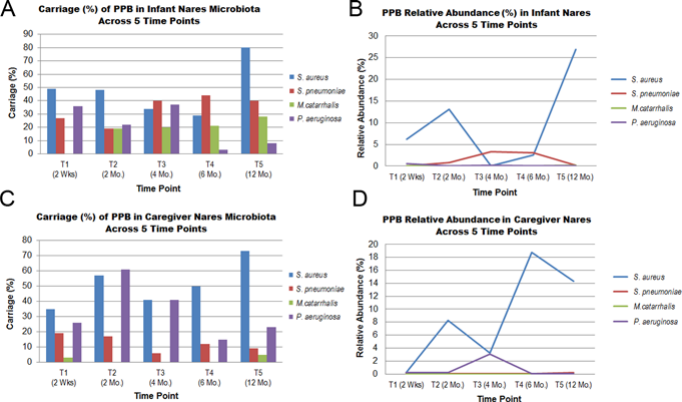
**Relative abundance (A, B) and carriage (C, D) of potentially pathogenic bacteria (PPB) in the nares of infants and their primary caregivers over the first year of life.** Relative abundance depicted represents the average values derived from each subject grouping at each sampling time point T1-T5 (range: 22–35 subjects per time point, in a possible maximum of (*n* = 40) study subject pairs). Carriage depicted represents the percentage of individuals carrying the organism at each time point.

Potentially pathogenic bacteria (PPB) were detectable in the anterior nare microbiomes sampled, including *Staphylococcus aureus* (detected in 29–80% subjects), *Streptococcus pneumoniae* (6–40%), *Moraxella catarrhalis* (0–28%), and *Pseudomonas aeruginosa* (3–61%) (Fig 2). Both *S*. *pneumoniae* (*p* = 0.0003, McNemar’s Exact) and *M*. *catarrhalis* (*p* = 6.1 x 10^−4)^ were consistently detected more prevalently in infants. Whereas *M*. *catarrhalis* was virtually undetectable in caregivers (range: 0–5% of subjects), approximately 20% (range 19–28%) of infants carried *M*. *catarrhalis* beginning at 1-month (T2) with sustained carriage throughout the remaining sampling time points, albeit with low relative abundance (0.1%). *S*. *pneumoniae* was detected across all sampling time points in both caregivers (range: 6–19% of subjects) and infants (range: 19–44%); however, approximately 20% (range 19–28%) of infants beginning at 2-months (T2) sustained *S*. *pneumoniae* carriage. During the summer time points (T3, T4) infant *S*. *pneumoniae* carriage was significantly higher than in caregivers (*p* = 0.001 for both). *P*. *aeruginosa* carriage ranged from 15–61% in caregivers, with variable prevalence over the course of the 5 sampling time points. During the first 3 sampling time points (up to 4-months of life), *P*. *aeruginosa* carriage ranged 22–37% in infants, but decreased drastically during the later sampling points (T4 (3%) and T5 (8%)).

The most frequently detected PPB was *S*. *aureus* in both infants (range: detected in 29–80% of subjects) and adult caregivers (35–73%). *S*. *aureus* was more frequently detected in infants at T5 than T4 (*p* = 0.00098). Adult caregivers showed a peak of *S*. *aureus* mean carriage abundance (18.8) at 6-months (T4) and second highest carriage abundance (14.4) at 12-months (T5); yet low overall relative abundance (<0.2) detected for *S*. *pneumoniae*. The peak carriage abundance detected for *S*. *aureus* in infants was sampling time point T5 (27.0%) (Panel A in Fig 2). Abundance of *S*. *aureus* and *S*. *pneumoniae* appeared inversely related in infants: relative abundance of *S*. *aureus* decreased in infants whereas *S*. *pneumoniae* relative abundance increased during the Canadian summer months (T3, T4) corresponding to 4 and 6 months of age (respectively).

### Nares microbiome diversity

To quantify and compare α-diversity, we calculated and compared the Shannon diversity index for all samples. Albeit with some subject variability noted, the mean Chao1 score for species richness across all time points was relatively consistent overall across the two study groups: 155.27 (range 39–391.3) in infants versus 143.59 (range: 49–413.5) in adult caregivers. Similarly, the mean Shannon diversity index for infants was 1.11 (range: 0.10–3.47), and in caregivers was 1.18 (range: 0.10–2.48). When diversity between caregivers and infants was assessed, we found no significant differences for most time points, except the Shannon diversity index in T1 (*p =* 0.005, Table 1).

**Table 1:** Richness and Diversity of Nares Microbiota in Infants and their Primary Caregivers.

Time point	No. of Participants	Richness Score^1^, mean ± SD	*p* value^2^	α-diversity Index^1^, mean ± SD	*p* value^2^
**2-Weeks (T1)**					
Caregivers	31	139.72 ± 68.00	0.9	**1.25 ± 0.53**	**0.005**
Infants	33	130.42 ± 68.70		**0.88 ± 0.61**	
**2-Months (T2)**					
Caregivers	23	151.79 ± 84.19	0.8	1.26 ± 0.48	0.15
Infants	27	146.63 ± 74.58		1.01 ± 0.58	
**4-Months (T3)**					
Caregivers	32	151.60 ± 91.23	0.97	1.21 ± 0.46	0.49
Infants	35	142.75 ± 64.66		1.16 ± 0.57	
**6-Months (T4)**					
Caregivers	34	122.35 ± 67.17	0.08	1.07 ± 0.62	0.57
Infants	34	155.50 ± 68.89		1.16 ± 0.57	
**12-Months (T5)**					
Caregivers	22	161.64 ± 71.28	0.59	1.15 ± 0.66	0.89
Infants	25	174.02 ± 83.62		1.33 ± 0.71	

SD = standard deviation

^1^ Richness was measured with the Chao1 score. α-diversity was measured with the Shannon diversity index

^2^ Wilcoxon signed-rank test.

**Fig 3:**
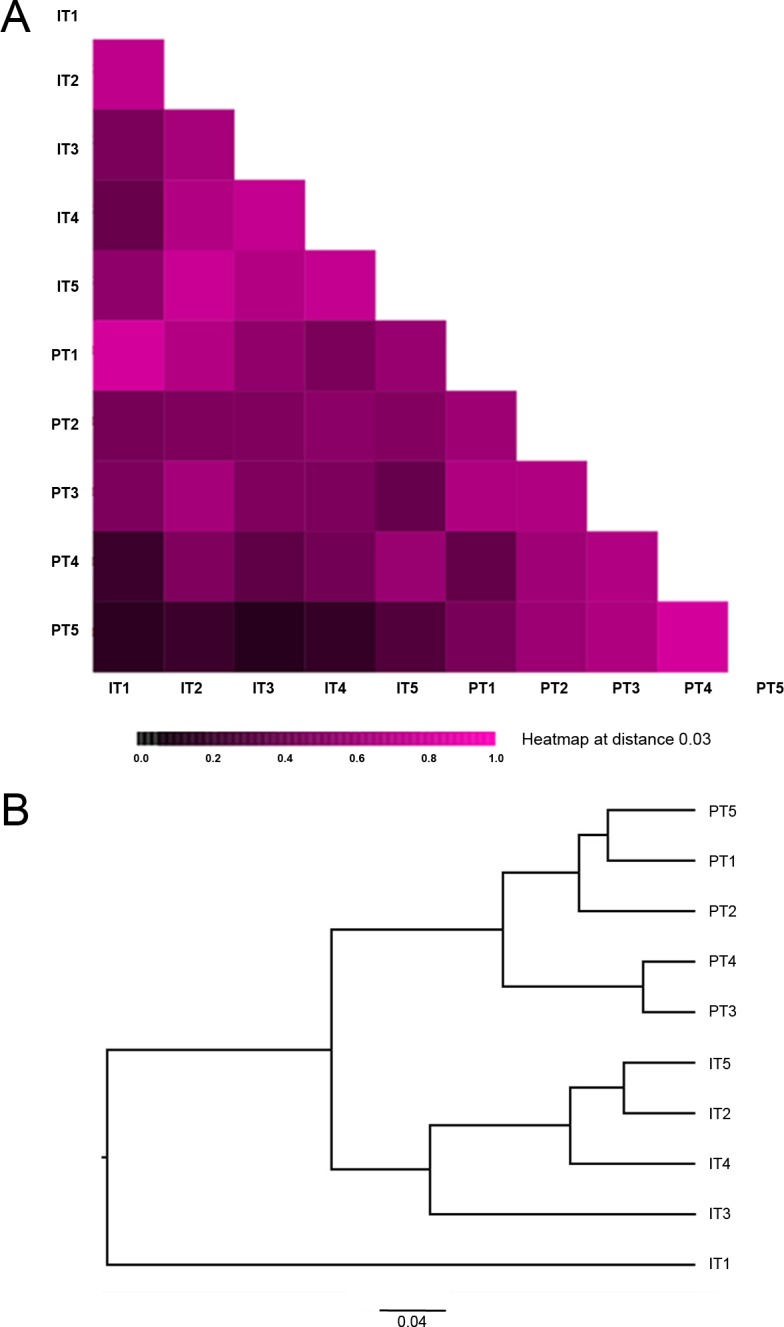
**Heatmap (A) and Phylogenetic Tree (B) of Morisita-Horn diversity indices between nares microbiota derived from infants (I) and their primary caregivers (P) at each time point (T1-T5).** A) Gradations in the shading intensity reflect the degree of similarity for Morisita-Horn diversity indices between sampling time points, with darkest hue intensities denoting most dissimilarity (approaching a Morisita-Horn diversity index value of 0.0) and brightest hue intensities denoting the most similarity (diversity index value approaching 1.0). B) Phylogenetic distance was calculated using the unweighted pair group method with arithmetic mean (UPGMA) for each time point, based on a random subsampling of 25,000 OTUs from all subjects per group. The scale bar depicts an inferred phylogenetic distance (Morisita-Horn diversity index) of 0.04 (no units).

Differences in microbiota also were assessed using β-diversity metrics. β-diversity was measured using the Morisita-Horn index (chosen for its ability to evaluate differences in populations of different sizes), as calculated in *mothur* between infants and their primary caregivers at each time point (Fig 3). For infants, the figure depicts the observed dissimilarity of the 2-week time point (T1) relative to all other time points (T2-T5) (Panel A in Fig 3). The tree demonstrates a seasonal trending of observed nares diversity in adult caregivers, with groupings of T1, T2 and T5 (winter) and T3 and T4 (summer) (Panel B in Fig 3).

The nares microbiome diversity for primary caregivers was relatively constant over all sampling time points (Table 1). In contrast, when we compared average relative diversity over time, infants at T1 (2 weeks of age) contained less diverse nares microbiota (Shannon diversity index) relative to infants at all later time points: T3 (4-months; *p =* 0.038, Wilcoxon Signed-Rank test), T4 (6-months; *p =* 0.037) and T5 (12-months; *p =* 0.018). The average species richness (Chao1) also increased in infants between T1 (2-weeks) and T5 (12-months) (*p =* 0.032) (Table 1).

### Longitudinal assessment of infant nare microbiome development

To investigate how microbiota of infants changed in their first year of life, we examined differences in the microbiota of our infant and caregiver cohort. As mentioned previously, the primary caregiver nares microbiome diversity was relatively constant over all sampling time points (Table 1). In adults, only *Acinetobacter* revealed significantly higher relative abundance at T1 sampling (2.1%) *vs*. T2 (0.06%), T3 (0) and T4 (0) (*p* < 0.0001, *q* < 0.0001 for all). In contrast, the predominant phyla and genera detected in infant nares varied more dramatically over time, with the previously described 2-week time point (T1) most dissimilar relative to all other time points (T2-T5) (Fig 3). Over the year of sampling, infants acquired a higher proportion of Firmicutes at the expense of Actinobacteria (Panel A in Fig 1; S5 Fig). A large proportion of the Firmicutes identified in this cohort were classified within the genus *Dolosigranulum*, along with *Staphylococcus* and *Streptococcus* (Panel B in Fig 1; S3 Fig); a large proportion of the Actinobacteria in this cohort were classified within the genus *Corynebacterium* and *Rhodococcus* (Panel B in Fig 1; S3 Fig). We then applied *mothur’s* implementation of *Metastats* with a 95% significance level to determine which OTUs significantly fluctuated over time. The most predominant taxa, genus *Staphylococcus*, virtually disappeared as a predominant genus at sampling points T3 (0.4% abundance) and T4 (1.9% abundance) in infants (4 and 6 months of age), but reappeared at the 12-month sampling point (T5–18.7% abundance) (Fig 1). Infants at 6-months (T4) had significantly greater *Staphylococcus* levels detected than in infants at 4-months (T3) and at 12-months (T5) (*p* < 0.00001, *q* < 0.00001 for both). Highest abundance of Gammaproteobacteria (class) [Proteobacteria (*Moraxella* (genus)] was noted at the T5 sampling (12-months of age) (*vs*. T1: *p* < 0.0001, T2: *p* = 0.00017, T3: *p* < 0.0001, T4: *p =* 0.001) (data not shown). When rudimentary multivariate analysis was conducted, in infants, *Staphylococcus* relative abundance was significantly higher at T5 *vs*. T3 (*p* = 0.001). *Corynebacterium* and *Dolosigranulum* carriage appeared to be affected by season: compared with T1 (winter) significant differences were noted at T3 (*p* <0.001, *Dolosigranulum*) and T4 (*p* <0.001 (*Dolosigranulum*), *p* = 0.0045 (*Corynebacterium*)), which are both summer time points (data not shown).

## Discussion

Little is known regarding nares microbiota in newborns and during early life, and there is an increasing body of evidence that host microbiomes modulate our innate resistance to pathogenic and opportunistic microbes. Baseline knowledge of nares microbiome development and influencing factors are important for promoting healthy infant development and to better recognize abnormal situations and prevent disease. To our knowledge, this study provides the first detailed prospective characterization of bacterial communities within the human nares microbiome in infants and their caregivers during the first year of life.

The majority of microbial community census studies to date have employed targeted 16S rRNA profiling. Our study employed an alternative profiling approach employing an internal universal target (UT) region within the protein-coding *cpn60* (also known as *hsp60* and *groEL*). *Cpn60* is a conserved molecular chaperone in bacteria and in eukaryotes [42]. Our justification for application of the internal *cpn60* universal target (UT) region (549–567 bp), which is amplifiable using degenerate broad-range (‘‘universal”) primers, is as follows: it has demonstrated sufficient resolution to discriminate closely related taxa at the species and subspecies level; only one copy is present per cell; and it can be more phylogenetically informative than the multi-copy 16S rRNA [30,42–45]. For example, *cpn60* UT sequences distinguish between *Staphylococcus* and *Macrococcus* (both known to be reside in human nares or oropharynx) when these are indistinguishable when employing 16S rRNA profiling [46]. As we anticipated potential for closely related taxa at the time of study initiation, we selected *cpn60* UT as the molecular barcode for our high resolution nares microbiome profiling. Regions from within the 16S rRNA and *cpn60* genes have since been subjected to assessment deploying the International Barcode of Life (iBOL) framework for evaluating molecular barcodes [42]. Within completed bacterial genomes in the public domain (representing 983 species from 21 phyla), *cpn60* was found to have a larger “barcode gap” (the largest difference between median pairwise inter- and intra-specific distances) relative to 16S rRNA regions examined. The published assessment again suggests *cpn60* as a preferred barcode for bacteria and that the *cpn60* UT is a robust target for species-level characterization [42].

Also when initiating our study, we were mindful that the presence of variable amounts of contaminating human genomic DNA within our collected samples might challenge the analysis. To circumvent this, we devised a processing protocol with the aim of preferentially enriching microbiome DNA relative to host DNA. Our method was based on selective cell lysis, which requires live bacterial cells and unfortunately led to low bacterial DNA recoveries. Compounded with our application of 454 pyrosequencing technology, with lower sequencing read yields inherent to the platform, our study generated fewer reads per subject than other studies might currently achieve with alternative sequencing technologies. Despite these caveats, when we applied our gene-based *cpn60* census profiling approach to the paired infant-caregiver cohort, we observed that the anterior nares microbiome is comprised primarily of three phyla (Actinobacteria, Firmicutes and Proteobacteria). The Human Microbiome Project (HMP) similarly detected a predominance of Actinobacteria and Firmicutes in healthy adult nares, with smaller relative abundances for Proteobacteria, Cyanobacteria and Bacteroides [47]. Differences in Bacteroides and Cyanobacteria detected levels in the HMP versus our study are most likely attributed to the employment of distinct taxonomic target barcodes, as the cpnDB database remains less comprehensive for taxonomic assignment than those available for 16S rRNA; high quality read yields for each study likely also played a role. We observed no consistent temporal trending for relative abundances of phyla or genera within adult individuals; furthermore, individual subjects predominantly carrying Actinobacteria during one time point often were detected with a high abundance of Firmicutes at the next sampling (S2 Fig). These findings are actually congruent with the HMP, in which adult nare samplings at different times (30–359 days) were only weakly correlated [13] and with more recent data from Flores *et al*. showing that temporal microbiome variability is personalized (i.e., subject specific) [4].

The anterior nare “core” microbiome (defined for the purpose of this study as taxa present at >0.1% abundance in at least 50% of study subjects (both infants and adult caregivers) during at least one time point [48,49]) was determined to be comprised of genera *Corynebacterium*, *Rhodococcus*, *Staphylococcus*, *Dolosigranulum*, *Streptococcus*, *Moraxella* (infants only), and the classes Gammaproteobacteria, and Actinobacteria (adult caregivers only) (Tables A and B in S5 File). Studying healthy adult nares, the HMP similarly described *Propionibacterium*, *Corynebacterium*, *Staphylococcus*, and *Moraxella* as the most predominant genera, with less abundant *Streptococcus*, *Bacteroides*, *Haemophilus*, *Prevotella*, *Veillonella*, and *Lactobacillus* [13]. Albeit a distinct anatomical site from our nares study, profiling of the nasopharyngeal (NP) microbiome in a prospective infant cohort also revealed the NP samples to be similarly dominated by six common genera, namely *Haemophilus*, *Streptococcus*, *Moraxella* (each more common in “infection” samples), and *Staphylococcus*, *Alloiococcus* and *Corynebacterium* (each more common in “healthy” samples) [50].

High inter-subject variability was observed, with large Chao1 richness and Shannon diversity index ranges (Table 1). In addition, inter-subject diversity [represented as the total number of OTUs present in each group (S4 Fig)] was higher for infants than for caregivers, while individual infants had lower intra-subject diversity measures than caregivers (Table 1). Lemon *et al*. [51] determined the average Chao1 richness for anterior nare samplings from a limited number (*n* = 7) of healthy adults by sequencing 16S rRNA gene clone libraries. Their average Chao1 richness value (50 ±7.2) was nearly 3-fold lower than our measurement (143.6) in adult caregivers; however, Lemon and colleagues had analyzed only 141–161 cloned reads per subject whereas we analyzed more than 1000 high quality sequenced reads per sample. Meanwhile, average Shannon diversity indices for anterior nares were determined by Oh *et al*. as 2.3 for pre-pubescent children and 1.9 for teenagers [16], and 1.7 in healthy adults by the HMP (study had extensive inter-subject variability) [14]. The average Shannon diversity values for our study were slightly lower, at 1.1 (infants) and 1.2 (caregivers) (Table 1). Inflated 16S rRNA-based diversity estimates can be attributed to the presence of multiple 16S rRNA gene copies present in bacterial chromosomes [32,52]. Additionally, a time-limited, linear augmentation step (multiple displacement amplification (MDA)) was used in our study to enrich the variable biomass-containing specimen templates prior to *cpn60* UT amplification, whereas the HMP and other studies applied a 16S rRNA target amplicon-based approach free of MDA. Additional differences in sample collection, processing and data analyses; dissimilarites across study populations (socioeconomic, climate, *etc*.) and natural variability in the nares [53] also may have contributed to diversity assessment discrepancies between studies.

We found relatively consistent nares community composition and diversity in adult primary caregivers over the study’s five sampling timepoints (Table 1). In contrast, both the average Chao1 and Shannon diversity indices consistently increased throughout the sampling periods providing evidence to support the diversity of the infant nasal microbiome increases longitudinally over the course of the first year of life. This is consistent with several studies of the infant intestinal (gut) microbiota, which have found a similar increase of diversity during the first year of life [24,54]. Our present study also revealed distinct nare microbiome composition in infants relative to those of their primary caregivers. Although caregivers exhibited a broader degree of microbial diversity, the greatest similarity in microbial composition between infants and caregivers was detected at 2-weeks of infant age (T1). Such close association at the first sampling point (T1) was expected owing to close physical contact between infants and primary caregivers and minimal environmental exposures [55]. Our data raises the possibility of mother-to-child transfer of nares microbiota, as has been similarly found for the gut microbiome [56]. Throughout all subsequent time points (T2-T5), nares microflora diversity increased and the two study groups diverged in terms of overall composition to a maximum pair-wise divergence at the final time point of 12-months (T5). This is consistent with other studies in which infants were initially colonized by maternal microbes, followed by individualization of microbiota thereafter, for example as the infant reaches 1–3 years of age [22,23]. Studies also have found that cohabitation does not result in a convergence of microbiota [18,57].

Study participants were from Winnipeg, a Canadian a city with a continental climate and substantial seasonal temperature changes between summer (26°C high/13°C low) and winter (-11°C high/-21°C low) [58]. Seasonal trending was observed in this study for adult caregivers in nares microbiome composition. Composition of caregiver nares microbiota for both winter time points (T1 and T5) were most similar, as were both time points in the summer season (T3 and T4). Bogaert *et al*. demonstrated seasonal trending of nasopharyngeal flora in children (aged 18-months) during autumn/winter *vs*. spring [26]; whereas infants in our study had the fewest shared taxonomic groups between the two winter season time points (T1, T5). However, seasonality is confounded with infant developmental age in our study cohort, and age itself causes infant physiological, mobility and immune system changes that may influence microbiota.

This study showed variations in both PPB carriage and abundance between infants and their primary caregivers as well as temporal PPB trending. In infants, *Moraxella catarrhalis* carriage rates increased from 0 to 28% carriage over the course of the year, even though the relative PPB abundance levels remained generally very low. *M*. *catarrhalis* is a human-restricted unencapsulated Gram negative bacterium previously associated with both commensal colonization of the nasopharynx and pathogenicity in the respiratory tract and inner ear (otitis media) [59]. This observation is congruent with previous findings that nasopharyngeal carriage of microbial pathogens capable of causing acute otitis media (middle ear inflammation) increases throughout the first year of life [60,61]. Abundance of *Pseudomonas aeruginosa* was low in the majority of participants; however two adults carried it predominantly at the summer T3 sampling period.

According to a recent Childhood Asthma Study (CAS) based out of Perth, Australia and involving a prospective cohort of 234 children, early nasopharyngeal (NP) colonization of infants typically involved *Staphylococcus* or *Corynebacterium*, was later replaced with *Moraxella* or *Alloiococcus* [51]. High rates of *Staphyloccus aureus* nasal colonization in the first months of life have been reported [62,63], and *S*. *aureus* has the potential to cause severe invasive disease in the newborn [50,62,64]. In the CAS study, presence of *Staphylococcus* was found to decline rapidly with age, and other age-related patterns also were observed amongst healthy and infected participant samples [51]. In congruence, *S*. *aureus* carriage rates in our study ranged 29–80% in infants and 35–73% in caregivers (Fig 2). The HMP similarly found relatively similar (29%) *S*. *aureus* carriage rates in healthy adult nares [13]. Previous studies also have described negative associations between *S*. *aureus* and *Streptococcus pneumoniae* carriage in both anterior nares and the nasopharynx [48,65]. The current study also observed an inverse relationship between *S*. *aureus* and *S*. *pneumoniae* detection in infants; yet this inverse relationship was not seen in our adult caregiver cohort. Whereas *S*. *pneumoniae* was present in less than 20% of our adult caregivers and at low relative abundance levels, conversely, *S*. *pneumoniae* was detected in 19–44% of all infant samplings with a mean relative abundance of 3.3% (Fig 2). In infants, *S*. *aureus* carriage and abundance declined during T3 and T4, whereas comcomitant carriage of *S*. *pneumoniae* increased. During the final sampling (T5), elevated *S*. *aureus* carriage was again observed (both in relative abundance and number of pathogen-colonized individuals) whereas *S*. *pneumoniae* carriage (relative abundance) decreased (exclusively in infants). These observations concur with past studies finding inverse correlations between *S*. *pneumoniae* and *S*. *aureus* carriage in infants [66].

Although we found no significant differences when investigating metadata, we saw greater *Staphylococcus* abundance in infants that were delivered vaginally *vs*. caesarean section (data not shown), contrary to a recent study by Shilts *et al*. [67]. We also found, although again not statistically significant, higher Chao1 richness estimates for the nasal microbiome of caesarean-delivered infants than for those born vaginally (data not shown). In concordance with prior studies [50,68], we noted an inverse correlation between the phyla Firmicutes and Actinobacteria in the nares (Fig 1A; S5 Fig). In our cohort, a large proportion of Actinobacteria were attributable to the genus *Corynebacterium*; whereas the Firmicutes were predominantly associated with the potentially pathogenic genera *Staphylococcus* and *Streptococcus*, as well as *Dolosigranulum* and *Veillonella*. Indeed, the most frequently detected PPB was *Staphylococcus aureus* in both infants and adult caregivers. Although none have identified to which species, several studies have noted genus *Corynebacterium* as negatively correlated with *S*. *aureus* growth [69] and colonization [70], suggesting a possible inhibitory effect of *Corynebacterium* on *Staphylococcus* growth. With potential infection risk as well as risk for transfer to others, the relative fluctuations of *S*. *aureus* potentially with *Corynebacterium* that we observed in our developing infant-adult caregiver cohort supports that further investigation is likely warranted.

Although this study described temporal and seasonal trending for differential abundance and frequency-based carriage of microbiota in infants *vs*. their primary caregivers, and also explored carriage of PPB, we recognize the importance of not overstepping concludable evidence [71]. Microbiomes are multifactorial and dynamic; there may be an unknown number of factors affecting the observed trends and variation described in this pilot study. Such factors should be an interesting area of future inquiry.

## Conclusions and Implications

Applying next-generation pyrosequencing with a *cpn60* gene-centric barcoding approach, we characterized the nares microbiota for developing infants throughout the first year of life alongside their primary caregivers, neither of which have been extensively studied. The findings demonstrate that such work is feasible and assisted in identifying temporal differences in infant nare microbiota that are likely attributable to developmental age. The collected evidence also support that temporal changes occur with respect to carriage of potentially pathogenic bacteria (PPBs). Expanded studies are needed to better ascertain which biological, environmental and socioeconomic factors influence anterior nares microflora composition, including influences of allergies and individual immune status, what contributions are made from parents, external caregivers and siblings, and to clearly define which factors most influence infant nares microbiome development. The accumulation of such baseline knowledge may some day help inform when individuals are predisposed to microbial infection.

## Supporting Information

S1 Fig Characteristics of infant-caregiver cohort study subjects. Information was obtained through a written questionnaire given to the primary caregiver at the first time point (T1–2 weeks) (Infant gender, ethnicity, delivery method, and antibiotics at delivery), or at all 5 time points (T1-T5) (pets, caregiver smoking habits, daycare attendance, number of household members, infant diet).
(TIF)

S2 Fig Relative abundance of the major phyla in the nares microbiome of infants (I) and their primary caregivers (C) across five time points during the first year of life. I-V correspond to– 2-weeks, 2-months, 4-months, 6-months, 12-months, or T1-T5, respectively.
(TIF)

S3 Fig Relative abundance of the major genera in the nares microbiome of infants (I) and their primary caregivers (C) across five time points during the first year of life. I-V correspond to– 2-weeks, 2-months, 4-months, 6-months, 12-months, or T1-T5, respectively.
(TIF)

S4 Fig Shared nares microbial taxa in infants and their primary adult caregivers across five time points during the first year of life. OTUs were clustered at 0.03 genetic distance (~97% sequence identity; such a level is considered to resolve to somewhere between genus and species level for *cpn60* UT. Venn diagrams were constructed in *mothur* applying the venn command, and were then redepicted in Microsoft Word and Abobe Illustrator.
(TIF)

S5 Fig Inverse correlation between percent relative abundance of Firmicutes and Actinobacteria for all nares samples ((slope (m) = -0.9279). Red line and inset equation represent the best fit line (*y = slope (x) + y intercept*). r^2^ = Coefficient of determination.
(TIF)

S1 File Consent Form and Participant Questionnaires. (PDF)

S2 File PCR Amplification of *cpn60* Universal Target (*cpn60* UT). **Text Preamble:** *Cpn60* Universal Target (*cpn60* UT) PCR Conditions. **Table A:** Oligonucleotides used for *cpn60* Universal Target (UT) PCR. **Table B:** Multiplex Identifier (MID) barcode sequences for Universal Target (UT) 454 pyrosequencing.
(PDF)

S3 File STROBE Statement—checklist of items that should be included in reports of observational studies. (PDF)

S4 File Frequency of dominant taxa (%) in nares swabs from healthy infants (Table A) and their primary caregivers (Table B) from 5 time points over the first year of life. Frequency of carriage of all taxa found in at least 10 participants at all time points.
(DOCX)

S5 File Median relative abundance of dominant taxa (Interquartile range in parentheses) as determined by number of quality-filtered (QF) 454 sequencing reads found in nares swabs from healthy infants (Table A) and their primary caregivers (Table B) from 5 time points during the first year of life. (DOCX)
