# Supplementary figures and images for: A Study of the Infant Nasal Microbiome Development over the First Year of Life and in Relation to Their Primary Adult Caregivers Using cpn60 Universal Target (UT) as a Phylogenetic Marker
Source: PLoS One. 2016 Mar 28;11(3):e0152493. doi: 10.1371/journal.pone.0152493 (PMC4809513; doi:10.1371/journal.pone.0152493)

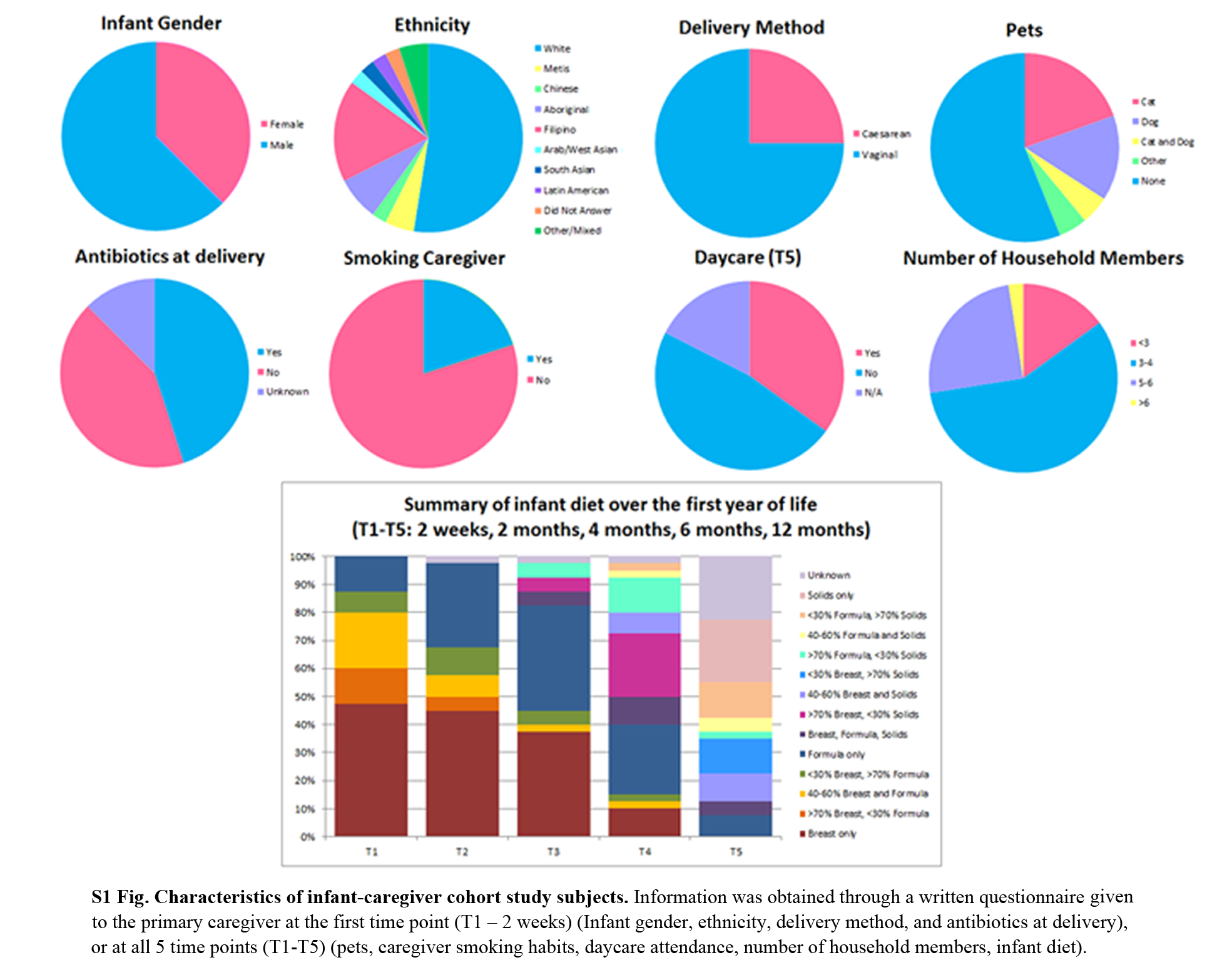

Supplement: S1 Fig — Information was obtained through a written questionnaire given to the primary caregiver at the first time point (T1–2 weeks) (Infant gender, ethnicity, delivery method, and antibiotics at delivery), or at all 5 time points (T1-T5) (pets, caregiver smoking habits, daycare attendance, number of household members, infant diet). (TIF) [file pone.0152493.s001.tif]

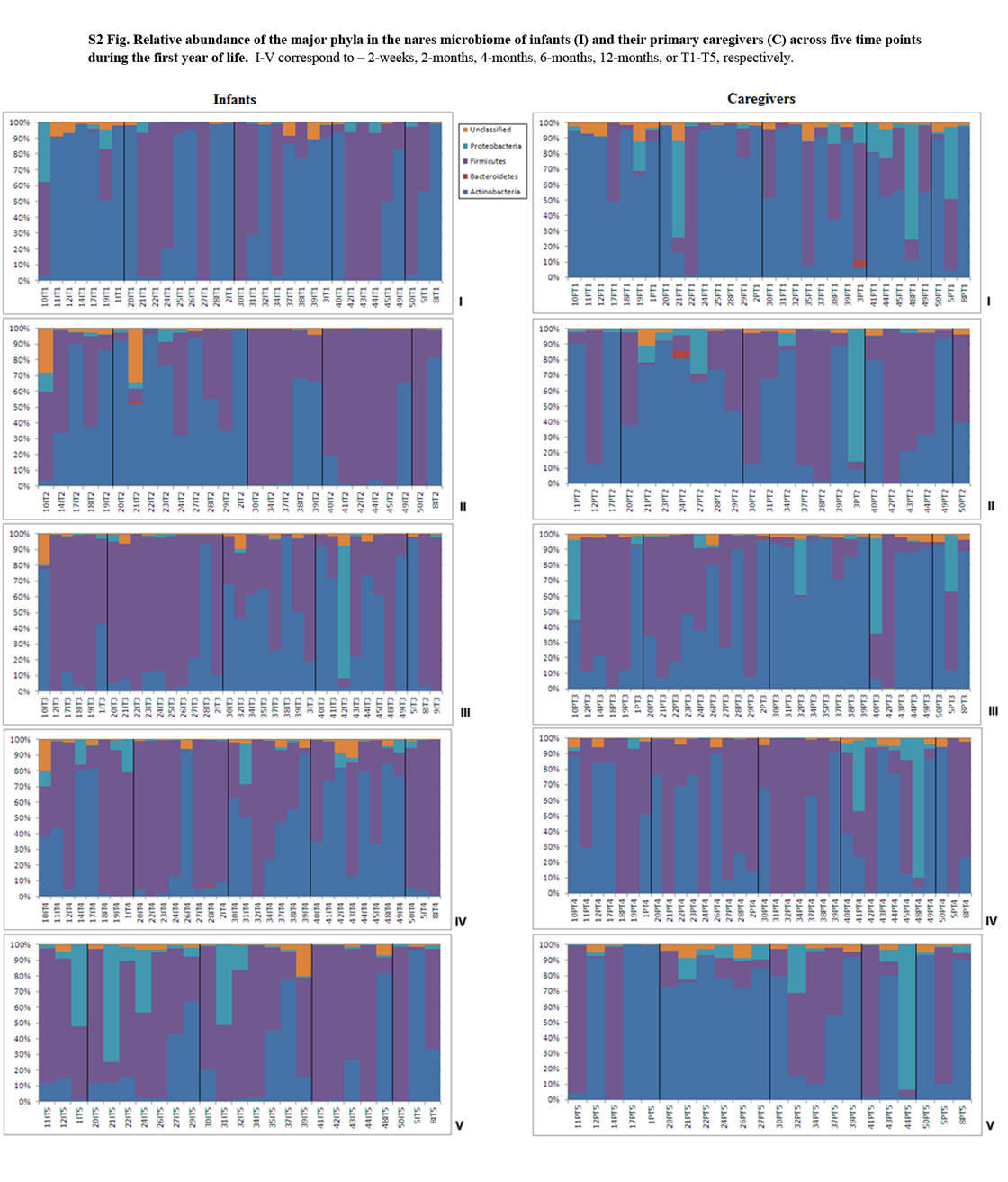

Supplement: S2 Fig — I-V correspond to– 2-weeks, 2-months, 4-months, 6-months, 12-months, or T1-T5, respectively. (TIF) [file pone.0152493.s002.tif]

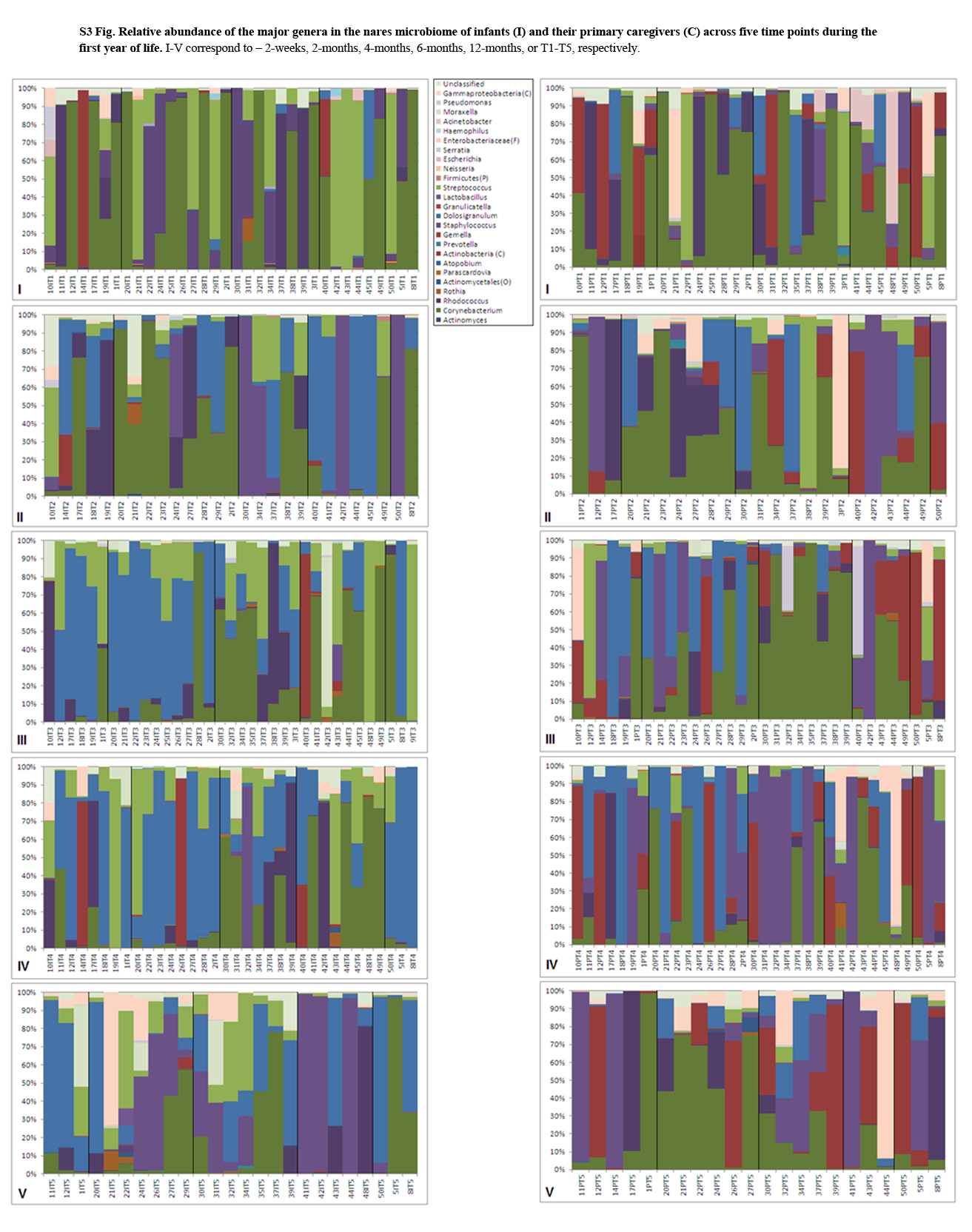

Supplement: S3 Fig — I-V correspond to– 2-weeks, 2-months, 4-months, 6-months, 12-months, or T1-T5, respectively. (TIF) [file pone.0152493.s003.tif]

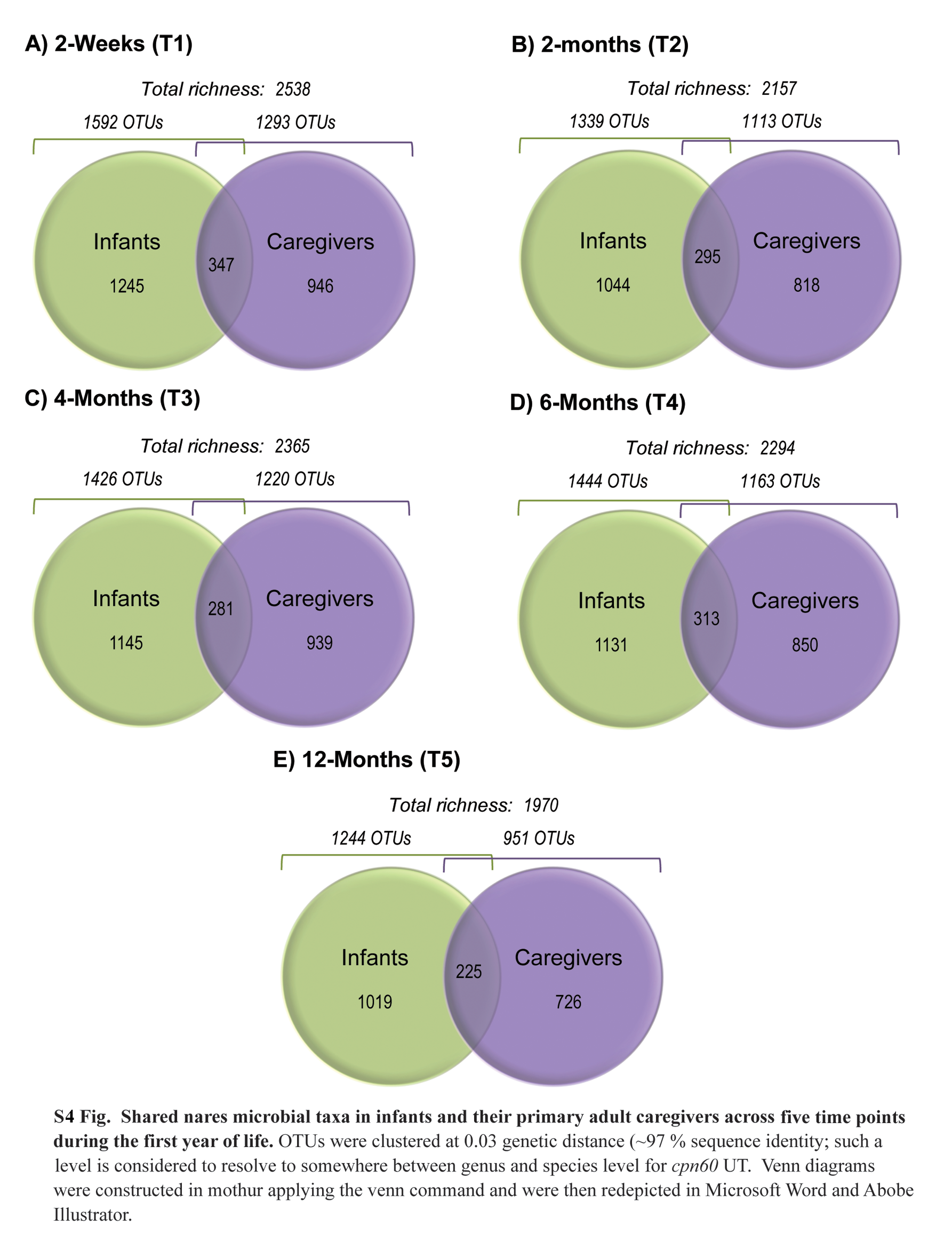

Supplement: S4 Fig — OTUs were clustered at 0.03 genetic distance (~97% sequence identity; such a level is considered to resolve to somewhere between genus and species level for cpn60 UT. Venn diagrams were constructed in mothur applying the venn command, and were then redepicted in Microsoft Word and Abobe Illustrator. (TIF) [file pone.0152493.s004.tif]

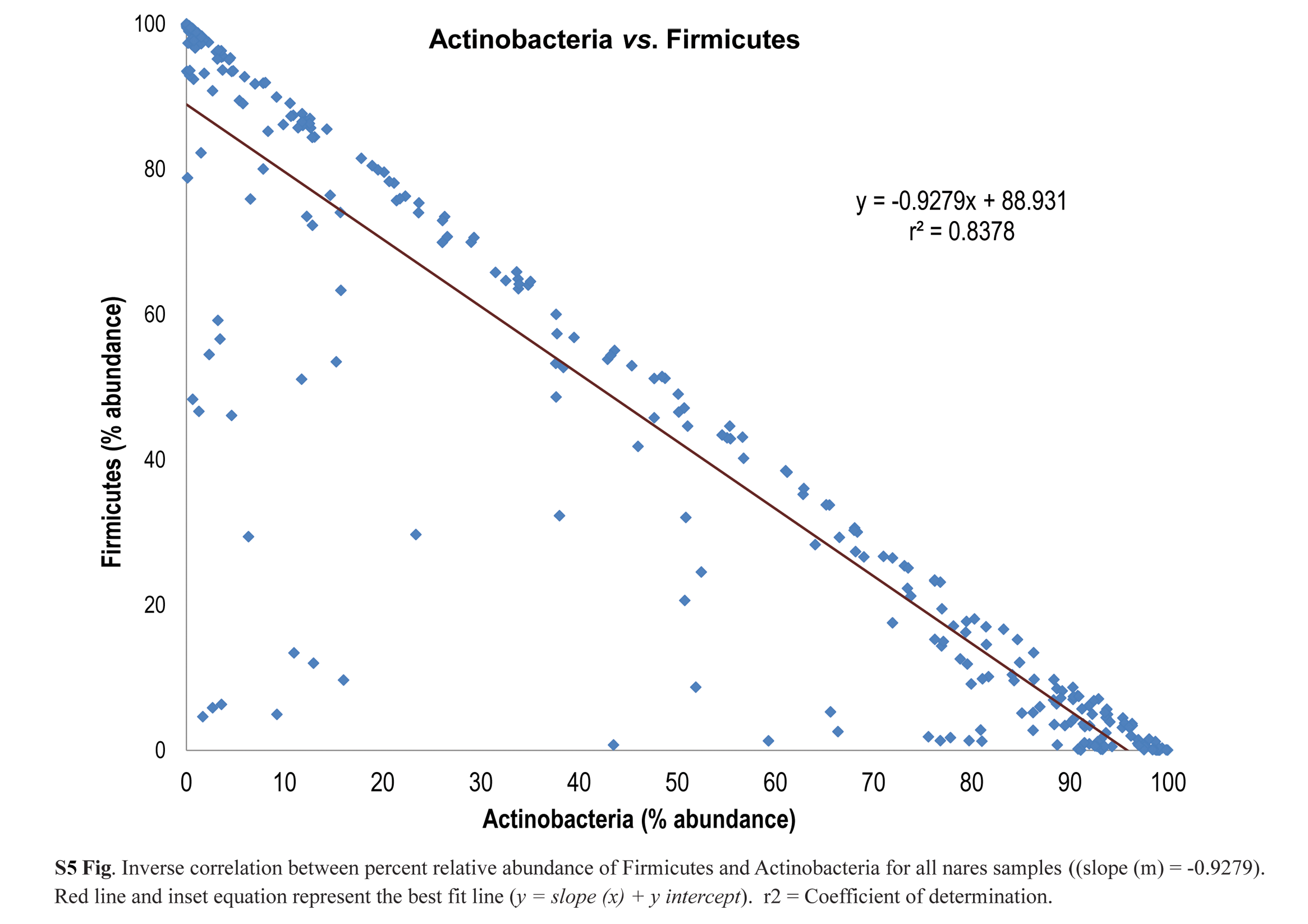

Supplement: S5 Fig — Red line and inset equation represent the best fit line (y = slope (x) + y intercept). r2 = Coefficient of determination. (TIF) [file pone.0152493.s005.tif]
