# Supplementary material for: A Study of the Infant Nasal Microbiome Development over the First Year of Life and in Relation to Their Primary Adult Caregivers Using cpn60 Universal Target (UT) as a Phylogenetic Marker
Source: PLoS One. 2016 Mar 28;11(3):e0152493. doi: 10.1371/journal.pone.0152493 (PMC4809513; doi:10.1371/journal.pone.0152493)
Supplement: S1 File — (PDF) [file pone.0152493.s006.pdf]

RESEARCH PARTICIPANT INFORMATION AND CONSENT FORM
FOR
NASAL MICROBIAL COMMUNITY STUDY

Title of Study: "Preliminary study on the nasal microbiome of infants".

Principal Investigators: Michael Mulvey, PhD, *National Microbiology Laboratory, Assistant professor of University of Manitoba, Department of Medical Microbiology*

Co-Investigators: Morag Graham, PhD, *National Microbiology Laboratory, Adjunct professor of University of Manitoba, Department of Medical Microbiology;* George Golding, PhD, *National Microbiology Laboratory; Fiona Fleming, MD, University of Manitoba, Adjunct professor of U of M, Department of Medical Microbiology;* Gary Van Domselaar PhD, *National Microbiology Laboratory; Sergio Fanella, MD, University of Manitoba;* Joanne Embree, MD, *University of Manitoba.*

Sponsor: *Federal Genomics Research and Development Initiative (GRDI)*

You and your child are being asked to participate in a Research Study. Please take your time to review this consent form and discuss any questions you may have with the study staff. You may take your time to make your decision whether to participate in this study and you may discuss it with your friends, family or (if applicable) your doctor before you make your decision. This consent form may contain words that you do not understand. Please ask the study staff to explain any words or information that you do not clearly understand. The study investigators do not receive any professional fees but do receive financial support to conduct this study.

Purpose of Study: Bacteria are normally present in all of us and provide numerous important and protective functions. This research study is being done to look at bacteria present inside the nose of infants throughout their early development (2 weeks to ~ 12 months of age). The purpose of this study is to obtain data on the normal, developing bacterial communities inside the nose of children and compare it to the bacteria found inside the nose of the primary caregivers.

Questions to be addressed include: (a) when and what bacteria appear inside the nose (from 2 weeks - ~12 months of age)? ; (b) do bacterial communities change with standard infant vaccinations or antimicrobial usage?; (c) how different are bacterial communities inside the nose of unrelated infants? (d) Are the bacteria similar in infants and primary care givers?

Study procedures: If you agree to participate in this study, you will have the following procedures: Sterile swabs will be used to collect nasal specimens from the primary care giver (yourself) and your child. These samples will be collected at your first visit to the clinic following the birth of your child, as well as your child's next scheduled appointments of ~2, 4, 6 and 12 months. From these samples, DNA from the germs will be extracted and will be tested to identify the types of bacteria residing inside the nose of the primary care giver (yourself) and your child. Once tested, any remaining nasal specimen will be destroyed. In the unlikely event that bacterial DNA remains after testing, it will be stored at the National Microbiology Laboratory in case a new genetic test is identified in the future. There will be no health consequences of sudden withdrawal from the study. You can stop participating at any time.

Risks and Discomforts: There are no known risks during the collection of the samples from inside the nose, but there may be slight irritation at the sampling sites.

Benefits: By participating in this study you will be providing information to the investigators that will improve our understanding of germs in the nose. There may or may not be direct benefit to you or your child from participating in this study. We hope the information learned from this study will provide future benefit to other children who are prone to childhood diseases caused by bacteria. A report summarizing findings from the study will be made available to you at the conclusion of the study, which will not contain any individual test results.

Costs: All the procedures, which will be performed as part of this study, are provided at no cost to you. The study investigators are not receiving any professional fees to conduct this study.

Payment for participation: Subjects who consent to participate in the study will not be compensated for their time and effort.

Confidentiality: Information gathered in this research study may be published or presented in public forums, however your name and other identifying information will not be used or revealed. Medical records that contain your identity will be treated as confidential in accordance with the Personal Health Information Act of Manitoba. Despite efforts to keep your personal information confidential, absolute confidentiality cannot be guaranteed. Your personal information may be disclosed if required by law. All study documents related to you will bear only your assigned patient number or code. The study team at the National Microbiology Laboratory (NML) will have access to that study code, but will not be able to link it to your personal information.
The data will be entered into a study computer, located at the NML, and only the study team from NML will have access to the data. Identifying information linking your identity will be kept on a separate study computer located at the Manitoba Clinic and only the treating physicians will have access to it. No agencies will receive this electronic data. Everyone involved at the clinic in the handling of medical records and personal health information has pledged the oath of confidentiality. The rest of the investigating team will never have access to any personal information and will not be able to link the assigned study code number with your personal health records.

The Health Canada Research Ethics Board may review records related to the study for quality assurance purposes.

All records will be kept in a locked secure area and only authorized Manitoba Clinic personal will have access to these records. If any of your medical/research records need to be copied to any of the above, your name and all identifying information will be removed. No information revealing any personal information such as your name, address or telephone number will leave the clinic.

Voluntary Participation/Withdrawal from the Study: Your decision to take part in this study is voluntary. You may refuse to participate or you may withdraw from the study at any time. Your decision not to participate or to withdraw from the study will not affect your care at this centre.

Questions

You are free to ask any questions that you may have about your treatment and your rights as a research participant. If any questions come up during or after the study or if you have a research-related injury, contact the principal investigator Dr. Michael Mulvey at (204) 789-2133.

For questions about your rights as a research participant, you may contact The University of Manitoba, Bannatyne Campus Research Ethics Board Office at (204) 789-3389.

Do not sign this consent form unless you have had a chance to ask questions and have received satisfactory answers to all of your questions.

Statement of Consent

I have read this consent form. I have had the opportunity to discuss this research study with …………………..and or his/her study staff. I have had my questions answered by them in language I understand. The risks and benefits have been explained to me. I believe that I have not been unduly influenced by any study team member to participate in the research study by any statements or implied statements. Any relationship (such as employer, supervisor or family member) I may have with the study team has not affected my decision to participate. I understand that I will be given a copy of this consent form after signing it. In the event that another individual has to bring in the child for a future scheduled appointment I am providing permission for samples to still be taken from the child. I understand that both my child and my participation in this study is voluntary and that I may choose to withdraw at any time. I freely agree to participate in this research study.

I understand that information regarding both my child and my personal identity will be kept confidential, but that confidentiality is not guaranteed. I authorize the inspection of any of my records that relate to this study by The University of Manitoba Research Ethics Board, for quality assurance purposes.

By signing this consent form, I have not waived any of the legal rights that I have as a participant in a research study.

Participant signature__________________________Date ____________________
(day/month/year)

Participant printed name: _____________________________

I, the undersigned, have fully explained the relevant details of this research study to the participant named above and believe that the participant has understood and has knowingly given their consent

Printed Name: __________________________Date ____________________
(day/month/year)

Signature: _____________________________

Initial Questionnaire

Please complete this questionnaire to the best of your knowledge. Feel free to ask Dr. Fleming (study doctor) or your pediatrician if you have any questions.

Study ID_____________________

Today's Date (yyyy/mm/dd) ________________

Date of Birth of Caregiver_____/____/____ (yyyy/mm/dd)
Gender of Caregiver Male Female

Date of Birth of Infant_____/____/____ (yyyy/mm/dd)
Gender of Infant Male Female

Self Claimed Ethnicity of Caregiver and Infant:

- ☐ Aboriginal
- ☐ Arab/West Asian (e.g. Armenian, Egyptian, Iranian, Lebanese, Moroccan)
- ☐ Black (e.g. African, Haitian, Jamaican, Somali)
- ☐ Chinese
- ☐ Filipino
- ☐ Japanese
- ☐ Korean
- ☐ Latin American
- ☐ South Asian
- ☐ South East Asian
- ☐ White (Caucasian)
- ☐ Other (*specify)* ______________________________
- ☐ Don't Know / Decline to Answer

Birth Delivery:

- ☐ Vaginal
- ☐ Caesarean
- ☐ Don't Know / Decline to Answer

Were antibiotics administered to the mother at delivery?

- ☐ Yes
- ☐ No
- ☐ Don't Know / Decline to Answer

Infant Feeding:

- ☐ Breast Milk (Approximate proportion %)___________
- ☐ Formula (Approximate proportion %)______________
- ☐ Solids (Approximate proportion %)________________
- ☐ Don't Know / Decline to Answer

Are you a smoker?

- ☐ Yes
- ☐ No
- ☐ Don't Know / Decline to Answer

Does anyone smoke inside your residence/car with you and/or your infant present?

	You	Infant
Daily	☐	☐
Weekly	☐	☐
Monthly	☐	☐
Less than Monthly	☐	☐
Never	☐	☐
Don't know/Decline to answer	☐	☐

Have you and/or your infant experienced any illness or infections in the last 2 months?

	You	Infant
Diarrhea	☐	☐
Ear Infection	☐	☐
Flu-Like Illness (ie. fever, aches, chills, cough, nausea)	☐	☐
Sinus (Nose) Infection	☐	☐
Throat Infection	☐	☐
Skin Infection	☐	☐
Urinary Tract Infection	☐	☐

Other (specify)____

Have you and/or your infant taken antibiotics in the past two months?

- ☐ No
- ☐ Yes--------------------------------->

☐ Caregiver (specify)____________________

____

☐ Infant (specify)________________________

____

☐ Don't Know / Decline to Answer

Do you or your infant have chronic skin or medical conditions?

	You	Infant
Allergies		
Environmental (ie. Pollen, cats)	☐	☐
Food	☐	☐
Medication (ie. Penicillin)	☐	☐
Other, please specify	☐	☐
Asthma	☐	☐
Cancer	☐	☐
Diabetes	☐	☐
Eczema/Psoriasis	☐	☐
Emphysema	☐	☐
Heart Disease	☐	☐
HIV/AIDS	☐	☐
Immunosuppressive therapy	☐	☐
Kidney Disease	☐	☐
Liver Disease	☐	☐

Other chronic conditions (specify)____

How many people are in your household?

- ☐ < 3
- ☐ 3 to 4
- ☐ 5 to 6
- ☐ > 6
- ☐ Don’t Know
- ☐ Decline to Answer

Do you have any household pets?

- ☐ No
- ☐ Yes (specify)_______________________
- ☐ Don’t Know
- ☐ Decline to Answer

Follow-up Questionnaire

Please complete this questionnaire to the best of your knowledge. Feel free to ask Dr. Fleming (study doctor) or your pediatrician if you have any questions.

*** If you are not the primary caregiver enrolled in this study - please forward this questionnaire to them. Please see the receptionist for a prepared envelope to return the completed form.**

Study ID_____________________

Today's Date (yyyy/mm/dd)________________

Infant Vaccinations:

- ☐ DaPTP (Diphtheria, acellular Pertussis, Tenanus, Polio)
- ☐ Hib (Haemophilus Influenzae B)
- ☐ MMR (Measles, Mumps, Rubella)
- ☐ PCV (Pneumococcal conjugate)
- ☐ MC (Meningococcal conjugate)
- ☐ Flu (Influenza)

Infant Feeding:

- ☐ Breast Milk (Approximate proportion %)__________
- ☐ Formula (Approximate proportion %)_____________
- ☐ Solids (Approximate proportion %)_______________
- ☐ Don't Know / Decline to Answer

Are you a smoker?

- ☐ Yes
- ☐ No
- ☐ Don't Know / Decline to Answer

Does anyone smoke inside your residence/car with you and/or your infant present?

	You	Infant
Daily	☐	☐
Weekly	☐	☐
Monthly	☐	☐
Less than Monthly	☐	☐
Never	☐	☐
Don't know/Decline to answer	☐	☐

Do you have any household pets?

- ☐ No
- ☐ Yes (specify)______________________
- ☐ Don't Know/Decline to Answer

Have you and/or your infant experienced any recent illness or infections within the past 2 months?

	You	Infant
Diarrhea	☐	☐
Ear Infection	☐	☐
Flu-like Illness (fever, aches, chills, cough, nausea)	☐	☐
Sinus (Nose) Infection	☐	☐
Throat Infection	☐	☐
Skin Infection	☐	☐
Urinary Tract Infection	☐	☐

Other (specify)____

Antibiotic usage for you and/or your infant within the past 2 months?

- ☐ No
- ☐ Yes----------------------------------> ◻ Caregiver (specify)___________________
  ____
  ◻ Infant (specify)______________________
  ____
  ◻ Don't Know / Decline to Answer
- ☐ Decline to Answer

Do you or your infant have chronic skin or medical conditions?

	You	Infant
Allergies		
Environmental (ie. Pollen, cats)	☐	☐
Food	☐	☐
Medication (ie. Penicillin)	☐	☐
Other, please specify	☐	☐
Asthma	☐	☐
Cancer	☐	☐
Diabetes	☐	☐
Eczema/Psoriasis	☐	☐
Emphysema	☐	☐
Heart Disease	☐	☐
HIV/AIDS	☐	☐
Immunosuppressive therapy	☐	☐
Kidney Disease	☐	☐
Liver Disease	☐	☐

Other chronic conditions (specify)____

Does your infant attend daycare?

- ☐ No
- ☐ Yes
- ☐ Don't Know/Decline to Answer
