# Supplementary material for: A Study of the Infant Nasal Microbiome Development over the First Year of Life and in Relation to Their Primary Adult Caregivers Using cpn60 Universal Target (UT) as a Phylogenetic Marker
Source: PLoS One. 2016 Mar 28;11(3):e0152493. doi: 10.1371/journal.pone.0152493 (PMC4809513; doi:10.1371/journal.pone.0152493)
Supplement: S2 File — Text Preamble: Cpn60 Universal Target (cpn60 UT) PCR Conditions. Table A: Oligonucleotides used for cpn60 Universal Target (UT) PCR. Table B: Multiplex Identifier (MID) barcode sequences for Universal Target (UT) 454 pyrosequencing. (PDF) [file pone.0152493.s007.pdf]

**Supplementary S2 File. PCR Amplification of *cpn60* Universal Target (*cpn60* UT).**

**Supplementary S2 Text Preamble:** *Cpn60* Universal Target (*cpn60* UT) PCR Conditions.

Amplification of *cpn60* UT was achieved using a standard PCR method with a 1:1 ratio of each set of LibL *cpn60* UT fusion primers (P279/P280 and P1612/P1613). Primers P279 and P1612 contained MID tag sequences to allow for identification during a 454 GS FLX sequencing run. There were 24 different MID-tagged primer sets used in total. PCR amplification was performed in either a Tetrad PTC-225 thermocycler or a PTC-200 thermocycler (Bio-Rad, Mississauga, Canada). Negative controls containing no DNA template (NTC controls) were included for all sets of PCR reactions. Positive controls were not included in order to prevent possible contamination.

PCR reactions were performed using 12.5 µl FailSafe PCR Premix G (Epicentre, Madison, USA), 2.5 µl 10x primer mix (containing 2 µM of each primer), 0.25 µl MTP *Taq* DNA Polymerase (Sigma-Aldrich, St. Louis, USA), 8.75 µl RT-PCR Grade water (Ambion, Austin, USA) and 1 µl of linearly augmented whole-genome template DNA. The PCR cycling program consisted of a 95$^{o}$C initial denaturation step, followed by 27 cycles of 94$^{o}$C for 30 seconds, annealing temperature for 1 minute, and 72$^{o}$C for one minute. This was followed by a final elongation step at 72$^{o}$C for 7 minutes and a final incubation at 4$^{o}$C. Three annealing temperatures (44$^{o}$C, 49$^{o}$C, 54$^{o}$C) were used to ensure greater amplification coverage over the broad range of GC+ contents for the various organism templates expected to be present. PCR reactions were performed in triplicate for each of three different annealing temperatures, with a total of nine reactions combined together for optimal yield and GC+ coverage.

**Supplementary S2 Table A:** Oligonucleotides used for *cpn60* Universal Target (UT) PCR

| Name | Oligonucleotide Sequence (5'-3')* |
|---|---|
| P279 | CGTATCGCCTCCCTCGCGCCA**TCAG**nnnnnnnnnnG AIIIIGCIGGIGAYGGIACIACIAC |
| P280 | CTATGCGCCTTGCCAGCCCGC**TCAG**YKIYKITCI CCRAAICCIGGIGCYTT |
| P1612 | CGTATCGCCTCCCTCGCGCCA**TCAG**nnnnnnnnnnG AIIIIGCIGGYGACGGYACSACSAC |
| P1613 | CTATGCGCCTTGCCAGCCCGC**TCAG**CGRCGRTC RCCGAAGCCSGGIGCCTT |

*Degenerate nucleotides indicated by standard IUPAC ambiguity codes;
I = deoxyinosine ('universal pairing base'); n=MID barcode sequence

**Supplementary S2 Table B:** Multiplex Identifier (MID) barcode sequences for Universal Target (UT) 454 pyrosequencing.

| Name | Sequence |
|---|---|
| **MID1** | ACGAGTGCGT |
| **MID2** | ACGCTCGACA |
| **MID3** | AGACGCACTC |
| **MID4** | AGCACTGTAG |
| **MID5** | ATCAGACACG |
| **MID6** | ATATCGCGAG |
| **MID7** | CGTGTCTCTA |
| **MID8** | CTCGCGTGTC |
| **MID9** | TAGTATCAGC |
| **MID10** | TCTCTATGCG |
| **MID11** | TGATACGTCT |
| **MID12** | TACTGAGCTA |
| **MID13** | CATAGTAGTG |
| **MID14** | CGAGAGATAC |
| **MID15** | ATACGACGTA |
| **MID16** | TCACGTACTA |
| **MID17** | CGTCTAGTAC |
| **MID18** | TCTACGTAGC |
| **MID19** | TGTACTACTC |
| **MID20** | ACGACTACAG |
| **MID21** | CGTAGACTAG |
| **MID22** | TACGAGTATG |
| **MID23** | TACTCTCGTG |
| **MID24** | TAGAGACGAG |
