# Supplementary material for: A Study of the Infant Nasal Microbiome Development over the First Year of Life and in Relation to Their Primary Adult Caregivers Using cpn60 Universal Target (UT) as a Phylogenetic Marker
Source: PLoS One. 2016 Mar 28;11(3):e0152493. doi: 10.1371/journal.pone.0152493 (PMC4809513; doi:10.1371/journal.pone.0152493)
Supplement: S4 File — Frequency of carriage of all taxa found in at least 10 participants at all time points. (DOCX) [file pone.0152493.s009.docx]

**S4 File Table A**: Frequency of dominant* taxa (%) in nares swabs from healthy infants from 5 time points throughout the first year of life

| Time point | 2 Weeks (T1) | 2 Months (T2) | 4 Months (T3) | 6 Months (T4) | 12 Months (T5) |
| --- | --- | --- | --- | --- | --- |
| *N* | 33 | 27 | 35 | 34 | 25 |
| **Actinobacteria** |  |  |  |  |  |
| *Actinomyces* | 0 (0) | 2 (7) | 2 (6) | 1 (3) | 3 (12) |
| *Corynebacterium* | 31 (94) | 26 (96) | 35 (100) | 34 (100) | 24 (96) |
| *Rhodococcus* | 26 (79) | 16 (59) | 25 (71) | 22 (65) | 15 (60) |
| *Rothia* | 6 (18) | 5 (19) | 15 (43) | 17 (50) | 14 (56) |
| Actinomycetales(Order) | 18 (55) | 18 (67) | 19 (54) | 18 (53) | 11 (44) |
| *Propionibacterium* | 1 (3) | 2 (7) | 1 (3) | 1 (3) | 2 (8) |
| *Bifidobacterium* | 2 (6) | 2 (7) | 0 (0) | 1 (3) | 3 (12) |
| Actinobacteria (Class) | 21 (64) | 17 (63) | 17 (49) | 17 (50) | 13 (52) |
| **Bacteroidetes** |  |  |  |  |  |
| *Prevotella* | 1 (3) | 2 (7) | 3 (9) | 4 (12) | 6 (24) |
| **Firmicutes** |  |  |  |  |  |
| *Gemella* | 7 (21) | 1 (4) | 2 (6) | 5 (15) | 3 (12) |
| *Staphylococcus* | 28 (85) | 22 (81) | 30 (86) | 22 (65) | 24 (96) |
| *Dolosigranulum* | 23 (70) | 26 (96) | 30 (86) | 31 (91) | 24 (96) |
| *Granulicatella* | 0 (0) | 0 (0) | 6 (17) | 9 (26) | 8 (32) |
| *Lactobacillus* | 3 (9) | 1 (4) | 0 (0) | 9 (26) | 3 (12) |
| *Streptococcus* | 28 (85) | 25 (93) | 35 (100) | 32 (94) | 25 (100) |
| *Anaerococcus* | 0 (0) | 0 (0) | 0 (0) | 0 (0) | 0 (0) |
| *Finegoldia* | 1 (0) | 0 (0) | 1 (3) | 0 (0) | 0 (0) |
| *Peptoniphilus* | 2 (0) | 0 (0) | 0 (0) | 0 (0) | 0 (0) |
| Clostridiales(Order) | 1 (3) | 0 (0) | 1 (3) | 1 (3) | 0 (0) |
| *Veillonella* | 0 (0) | 0 (0) | 4 (11) | 4 (12) | 0 (0) |
| Unclassified Firmicutes | 6 (18) | 4 (15) | 2 (6) | 9 (26) | 4 (16) |
| **Proteobacteria** |  |  |  |  |  |
| *Ralstonia* | 2 (6) | 0 (0) | 1 (3) | 2 (6) | 2 (8) |
| *Neisseria* | 2 (6) | 1 (4) | 0 (0) | 3 (9) | 4 (16) |
| *Escherichia* | 2 (6) | 2 (7) | 0 (0) | 1 (3) | 2 (8) |
| *Serratia* | 3 (9) | 2 (7) | 2 (6) | 1 (3) | 2 (8) |
| *Haemophilus* | 1 (3) | 2 (7) | 2 (6) | 1 (3) | 0 (0) |
| *Acinetobacter* | 9 (26) | 3 (11) | 0 (0) | 0 (0) | 3 (12) |
| *Moraxella* | 5 (15) | 13 (48) | 18 (51) | 19 (56) | 18 (72) |
| *Pseudomonas* | 15 (45) | 6 (22) | 15 (43) | 4 (12) | 8 (32) |
| Xanthomonadaceae(Family) | 2 (6) | 1 (4) | 0 (0) | 1 (3) | 2 (8) |
| Gammaproteobacteria(Class) | 18 (55) | 14 (52) | 10 (29) | 22 (65) | 25 (100) |
| Unclassified Proteobacteria | 3 (9) | 0 (0) | 3 (9) | 1 (3) | 1 (4) |
| **Unclassified** | 26 (79) | 24 (89) | 35 (100) | 33 (97) | 21 (84) |
|  | | | | |  |

*Taxa observed in greater than 10 individuals across all time points

**S4 File Table B**: Frequency of dominant* taxa (%) in nares swabs from healthy caregivers from 5 time points over the course of one year

| Time point | 2 Weeks (T1) | 2 Months (T2) | 4 Months (T3) | 6 Months (T4) | 12 Months (T5) |
| --- | --- | --- | --- | --- | --- |
| N | 31 | 23 | 32 | 34 | 22 |
| **Actinobacteria** |  |  |  |  |  |
| *Actinomyces* | 2 (6) | 0 (0) | 0 (0) | 0 (0) | 0 (0) |
| *Corynebacterium* | 31 (100) | 21 (91) | 31 (97) | 30 (88) | 22 (100) |
| *Rhodococcus* | 24 (77) | 16 (70) | 25 (78) | 18 (53) | 17 (77) |
| *Rothia* | 12 (39) | 8 (35) | 9 (28) | 6 (18) | 6 (27) |
| Actinomycetales (Order) | 23 (74) | 7 (30) | 15 (47) | 10 (29) | 9 (41) |
| *Propionibacterium* | 11 (35) | 7 (30) | 13 (41) | 14 (41) | 10 (45) |
| *Bifidobacterium* | 1 (3) | 1 (4) | 2 (6) | 1 (3) | 0 (0) |
| Actinobacteria (Class) | 26 (84) | 20 (87) | 27 (84) | 23 (68) | 20 (91) |
| **Bacteroidetes** |  |  |  |  |  |
| *Prevotella* | 2 (6) | 1 (4) | 1 (3) | 2 (6) | 0 (0) |
| **Firmicutes** |  |  |  |  |  |
| *Gemella* | 1 (3) | 1 (4) | 3 (9) | 3 (9) | 0 (0) |
| *Staphylococcus* | 26 (84) | 23 (100) | 32 (100) | 33 (97) | 21 (95) |
| *Dolosigranulum* | 19 (61) | 16 (70) | 22 (69) | 29 (85) | 19 (86) |
| *Granulicatella* | 4 (13) | 4 (17) | 2 (6) | 3 (9) | 2 (9) |
| *Lactobacillus* | 2 (6) | 0 (0) | 1 (3) | 1 (3) | 0 (0) |
| *Streptococcus* | 26 (84) | 23 (100) | 30 (94) | 32 (94) | 17 (77) |
| *Anaerococcus* | 3 (10) | 4 (17) | 4 (13) | 5 (15) | 0 (0) |
| *Finegoldia* | 3 (10) | 2 (9) | 3 (9) | 3 (9) | 1 (5) |
| *Peptoniphilus* | 5 (16) | 3 (13) | 7 (22) | 5 (15) | 3 (14) |
| Clostridiales (Order) | 1 (3) | 2 (9) | 1 (3) | 4 (12) | 1 (5) |
| *Veillonella* | 0 (0) | 1 (4) | 1 (3) | 2 (6) | 0 (0) |
| Unclassified Firmicutes | 4 (13) | 4 (17) | 3 (9) | 8 (24) | 2 (9) |
| **Proteobacteria** |  |  |  |  |  |
| *Ralstonia* | 5 (16) | 5 (22) | 2 (6) | 1 (3) | 2 (9) |
| *Neisseria* | 0 (0) | 1 (4) | 1 (3) | 0 (0) | 1 (5) |
| *Escherichia* | 1 (3) | 2 (9) | 1 (3) | 3 (9) | 3 (14) |
| *Serratia* | 8 (26) | 7 (30) | 6 (19) | 2 (6) | 4 (18) |
| *Haemophilus* | 0 (0) | 1 (4) | 2 (6) | 1 (3) | 1 (5) |
| *Acinetobacter* | 9 (29) | 3 (13) | 2 (6) | 0 (0) | 0 (0) |
| *Moraxella* | 10 (32) | 8 (35) | 9 (28) | 8 (24) | 5 (23) |
| *Pseudomonas* | 15 (48) | 14 (61) | 16 (50) | 8 (24) | 9 (41) |
| Xanthomonadaceae (Family) | 3 (10) | 3 (13) | 5 (16) | 0 (0) | 2 (9) |
| Gammaproteobacteria(Class) | 22 (71) | 14 (61) | 7 (22) | 22 (65) | 21 (95) |
| Unclassified Proteobacteria | 5 (16) | 2 (9) | 1 (3) | 5 (15) | 1 (5) |
| **Unclassified** | 31 (100) | 20 (87) | 30 (94) | 28 (82) | 19 (86) |
|  | | | | | |

*Taxa observed in greater than 10 individuals across all time points
