# Supplementary material for: A Study of the Infant Nasal Microbiome Development over the First Year of Life and in Relation to Their Primary Adult Caregivers Using cpn60 Universal Target (UT) as a Phylogenetic Marker
Source: PLoS One. 2016 Mar 28;11(3):e0152493. doi: 10.1371/journal.pone.0152493 (PMC4809513; doi:10.1371/journal.pone.0152493)
Supplement: S5 File — (DOCX) [file pone.0152493.s010.docx]

**S5 File Table A**: Median relative abundance of dominant taxa (Interquartile range in parentheses) found in nares swabs from healthy infants from 5 time points during the first year of life

|  | | | | | |
| --- | --- | --- | --- | --- | --- |
| Taxon* | 2 Weeks (T1) | 2 Months (T2) | 4 Months (T3) | 6 Months (T4) | 12 Months (T5) |
| **Actinobacteria** | 83.2  (3.2-93.8) | 37.7  (2.8-78.8) | 21.1  (3.6-66.6) | 28.7  (4.5-70.6) | 13.0  (1.5-33.8) |
| *Corynebacterium* | 28.1  (0.6-92.5) | 16.6  (0.6-66.9) | 8.1  (0.5-53.3) | 2.4  (0.4-31.1) | 1.6  (0.1-20.7) |
| *Rhodococcus* | 0.2  (0.0-0.7) | 0.0  (0.0-7.8) | 0.2  (0.0-4.1) | 0.0  (0.0-3.1) | 0.0  (0.0-0.1) |
| *Rothia* | 0.0  (0.0-0.0) | 0.0  (0.0-0.0) | 0.0  (0.0-0.1) | 0.0  (0.0-0.1) | 0.0  (0.0-0.1) |
| Actinomycetales (Order) | 0.0  (0.0-0.1) | 0.0  (0.0-0.1) | 0.0  (0.0-0.1) | 0.0  (0.0-0.1) | 0.0  (0.0-0.1) |
| *Propionibacterium* | 0.0  (0.0-0.0) | 0.0  (0.0-0.0) | 0.0  (0.0-0.0) | 0.0  (0.0-0.0) | 0.0  (0.0-0.0) |
| Actinobacteria (Class) | 0.0  (0.0-0.1) | 0.0  (0.0-0.1) | 0.0  (0.0-0.1) | 0.0  (0.0-0.2) | 0.0  (0.0-0.1) |
| **Firmicutes** | 16.6  (0.7-90.8) | 56.6  (12.5-96.5) | 76.3  (28.5-92) | 69.1  (21.8-93.4) | 70.7  (48.3-86.0) |
| *Staphylococcus* | 1.5  (0.0-15.4) | 0.3  (0.0-4.1) | 0.0  (0.0-0.2) | 0.0  (0.0-0.2) | 0.8  (0.1-38.6) |
| *Dolosigranulum* | 0.1  (0.0-0.3) | 6.0  (0.4-52.2) | 29.6  (0.8-72.9) | 34.5  (2.4-72.0) | 10.5  (1.4-57.7) |
| *Streptococcus* | 0.3  (0.1-19.1) | 1.3  (0.1-7.3) | 12.6  (0.7-32.1) | 5.8  (0.9-23.3) | 4.6  (1.0-14.0) |
| **Proteobacteria** | 0.1  (0.0-0.6) | 0.1  (0.0-1.0) | 0.3  (0.0-0.8) | 0.5  (0.2-2.8) | 1.7  (0.6-5.9) |
| *Moraxella* | 0.0  (0.0-0.0) | 0.0  (0.0-0.1) | 0.0  (0.0-0.4) | 0.0  (0.0-0.3) | 0.3  (0.0-1.1) |
| *Pseudomonas* | 0.0  (0.0-0.0) | 0.0  (0.0-0.0) | 0.0  (0.0-0.1) | 0.0  (0.0-0.0) | 0.0  (0.0-0.0) |
| Gammaproteobacteria (Class) | 0.0  (0.0-0.2) | 0.0  (0.0-0.6) | 0.0  (0.0-0.0) | 0.2  (0.0-1.2) | 0.6  (0.3-3.9) |
| **Unclassified** | 0.3  (0.0-1.3) | 0.6  (0.1-1.4) | 0.6  (0.3-1.4) | 0.5  (0.2-2.4) | 0.6  (0.2-2.6) |
|  | | | | | |

*Taxa with an interquartile range of 0.0-0.0 for both groups during all time points are omitted

**S5 File Table B**: Median relative abundance of dominant taxa (Interquartile range in parentheses) found in nares swabs from primary caregivers from 5 time points over the course of one year

|  | | | | | | |
| --- | --- | --- | --- | --- | --- | --- |
| Taxon* | 2 Weeks (T1) | 2 Months (T2) | 4 Months (T3) | 6 Months (T4) | 12 Months (T5) | |
| **Actinobacteria** | 88.3  (49.9-95.5) | 65.6  (17.1-83.7) | 65.1  (16.3-91.5) | 27.7  (1.1-76.9) | 77.2  (11.8-91.2) | |
| *Corynebacterium* | 24.2  (4.1-73.9) | 21.0  (4.1-56.8) | 23.9  (2.4-61.6) | 3.3  (0.1-14.8) | 9.6  (2.5-41.1) | |
| *Rhodococcus* | 0.1  (0.0-5.4) | 0.2  (0.0-5.2) | 0.1  (0.0-1.6) | 0.0  (0.0-0.3) | 0.1  (0.0-0.3) | |
| *Rothia* | 0.0  (0.0-0.0) | 0.0  (0.0-0.0) | 0.0  (0.0-0.0) | 0.0  (0.0-0.0) | 0.0  (0.0-0.0) | |
| Actinomycetales (Order) | 0.0  (0.0-0.3) | 0.0  (0.0-0.0) | 0.0  (0.0-0.1) | 0.0  (0.0-0.0) | 0.0  (0.0-0.0) | |
| *Propionibacterium* | 0.0  (0.0-0.0) | 0.0  (0.0-0.0) | 0.0  (0.0-0.0) | 0.0  (0.0-0.1) | 0.0  (0.0-0.1) | |
| Actinobacteria (Class) | 0.6  (0.0-13.2) | 0.4  (0.1-13.3) | 1.5  (0.1-23.4) | 0.5  (0.0-21.9) | 1.1  (0.0-34.0) | |
| **Firmicutes** | 5.7  (1.1-41.6) | 25.1  (5.2-70.7) | 12.0  (3.5-74.1) | 49.9  (15.0-93.4) | 10.8  (2.2-51.0) | |
| *Staphylococcus* | 1.3  (0.1-3.8) | 1.2  (0.6-10.2) | 1.2  (0.2-11.8) | 5.0  (0.3-42.7) | 2.8  (0.4-30.4) | |
| *Dolosigranulum* | 0.0  (0.0-1.0) | 0.1  (0.0-13.6) | 0.2  (0.0-12.8) | 2.9  (0.1-28.8) | 0.2  (0.0-11.1) | |
| *Streptococcus* | 0.5  (0.0-1.9) | 1.0  (0.1-3.9) | 0.6  (0.0-1.9) | 0.3  (0.1-1.3) | 0.1  (0.0-1.2) | |
| **Proteobacteria** | 0.8  (0.2-7.9) | 0.6  (0.1-3.4) | 0.1  (0.0-1.3) | 0.1  (0.0-1.8) | 1.9  (0.3-7.0) | |
| *Moraxella* | 0.0  (0.0-0.0) | 0.0  (0.0-0.1) | 0.0  (0.0-0.0) | 0.0  (0.0-0.0) | 0.0  (0.0-0.0) | |
| *Pseudomonas* | 0.0  (0.0-0.1) | 0.0  (0.0-0.2) | 0.0  (0.0-0.1) | 0.0  (0.0-0.0) | 0.0  (0.0-0.1) | |
| Gammaproteobacteria (Class) | 0.2  (0.0-0.8) | 0.0  (0.0-1.9) | 0.0  (0.0-0.0) | 0.1  (0.0-1.0) | 1.4  (0.3-6.6) | |
| **Unclassified** | 1.3  (0.5-3.5) | 1.1  (0.4-2.5) | 1.4  (0.7-2.4) | 0.4  (0.1-4.0) | 1.4  (0.2-3.5) | |
|  | | | | | |  |

*Taxa with an interquartile range of 0.0-0.0 for both groups during all time points are omitted
